# The combination of multi-approach studies to explore the potential therapeutic mechanisms of imidazole derivatives as an MCF-7 inhibitor in therapeutic strategies

**DOI:** 10.3389/fchem.2023.1197665

**Published:** 2023-06-27

**Authors:** Maryam Rashid, Ayesha Maqbool, Nusrat Shafiq, Yousef A. Bin Jardan, Shagufta Parveen, Mohammed Bourhia, Hiba-Allah Nafidi, Rashid Ahmed Khan

**Affiliations:** ^1^ Synthetic and Natural Product Drug Discovery Laboratory, Department of Chemistry, Government College Women University Faisalabad, Faisalabad, Pakistan; ^2^ Department of Pharmaceutics, College of Pharmacy, King Saud University, Riyadh, Saudi Arabia; ^3^ Department of Applied Chemistry, Beijing Institute of Technology, Beijing, China; ^4^ Department of Chemistry and Biochemistry, Faculty of Medicine and Pharmacy, Ibn Zohr University, Laayoune, Morocco; ^5^ Department of Food Science, Faculty of Agricultural and Food Sciences, Laval University, Quebec City, QC, Canada; ^6^ Nuclear Institute for Agriculture and Biology (NIAB), Faisalabad, Pakistan

**Keywords:** breast cancer, drug discovery, imidazole, virtual screening, MCF-7

## Abstract

Breast cancer covers a large area of research because of its prevalence and high frequency all over the world. This study is based on drug discovery against breast cancer from a series of imidazole derivatives. A 3D-QSAR and activity atlas model was developed by exploring the dataset computationally, using the machine learning process of Flare. The dataset of compounds was divided into active and inactive compounds according to their biological and structural similarity with the reference drug. The obtained PLS regression model provided an acceptable *r* ^2^ = 0.81 and q^2^ = 0.51. Protein-ligand interactions of active molecules were shown by molecular docking against six potential targets, namely, TTK, HER2, GR, NUDT5, MTHFS, and NQO2. Then, toxicity risk parameters were evaluated for hit compounds. Finally, after all these screening processes, compound **C10** was recognized as the best-hit compound. This study identified a new inhibitor C10 against cancer and provided evidence-based knowledge to discover more analogs.

## Graphical Abstract

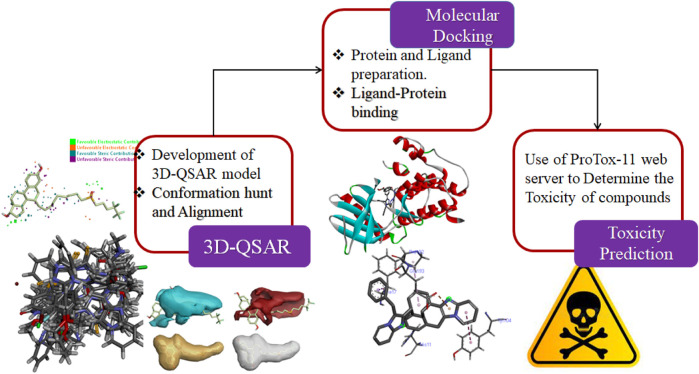

## Introduction

Cancer is the abnormal and uncontrolled growth of cells that is caused by the mutation of genes. This mutation may lead to an accelerated rate of cell division, so it is the major cause of death worldwide (Ali et al., 2017). A frequently occurring cancer in women is breast cancer and approximately 1 million women are affected by it every year*.* Obesity, consumption of alcohol, genetics, aging, menopause, diabetes mellitus (type 2), high estrogen levels, radiation exposure, smoking, menarche, sex, and physical activity are the major risk factors responsible for causing breast cancer (Ataollahi et al., 2015; Escala-Garcia et al., 2020; Anandan et al., 2022).

**FIGURE 1:**
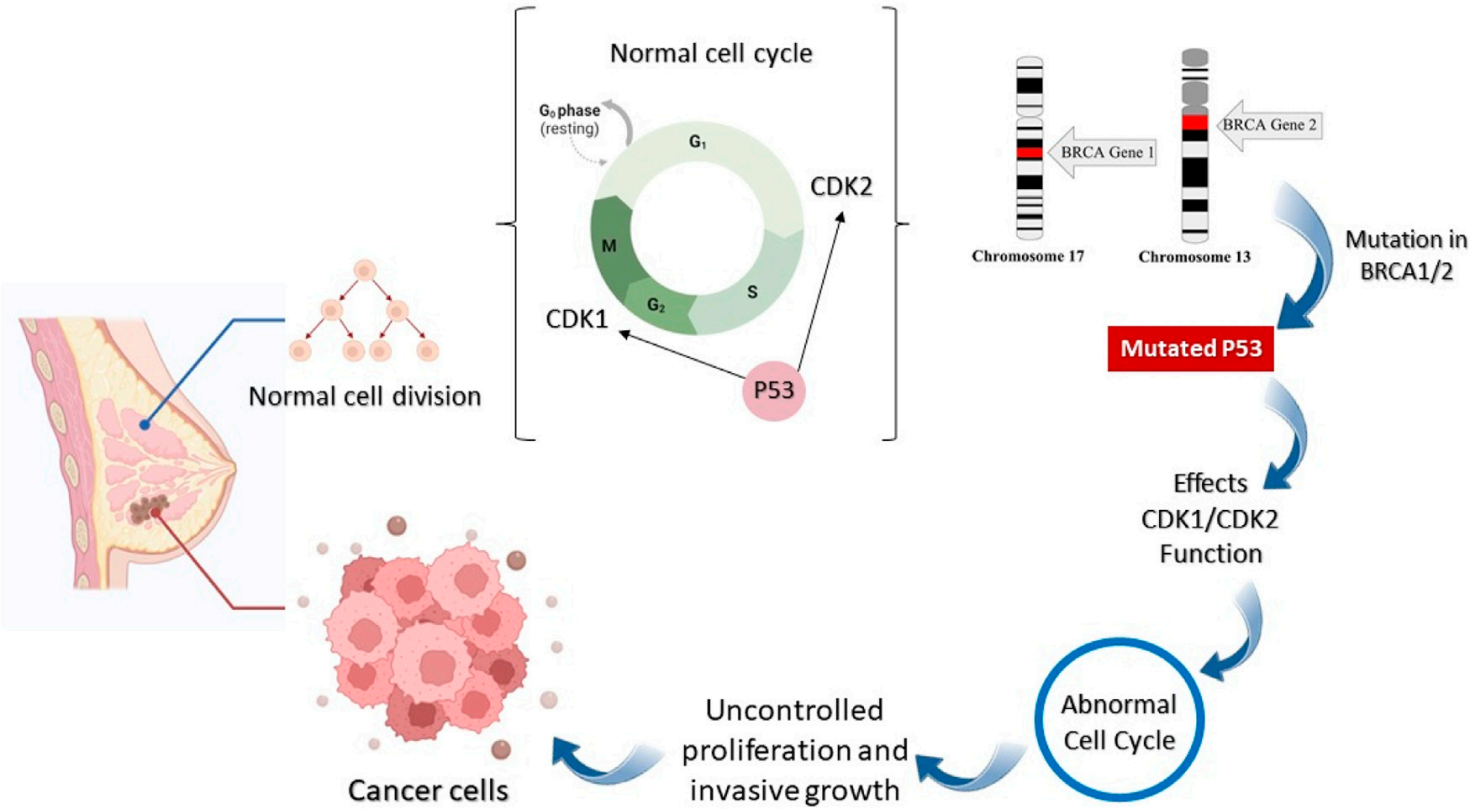
Anomalous amplification and mutation of genes for the initiation of tumor.

The genetic mutation causes the development and progression of breast tumors. Anomalous amplification and mutation of genes cause the initiation of tumors such as a mutation in the Breast Cancer gene (BRCA1/2), RB Transcriptional Corepressor 1 (RB1), Human epidermal growth factor receptor 2 (HER2), Fragile Histidine Triad Diadenosine Triphosphatase (FHIT), tumor protein P53, Epidermal Growth Factor Receptor (EGFR), extracellular signal-regulated kinase (ERK), Mitogen-activated protein kinase (MEK), and Rat sarcoma (Ras) genes that can lead to breast cancer (Figure 1) (Dickson, 1990; Sun et al., 2017; Lakshmithendral et al., 2019a).

Endogenous estrogen and exogenous estrogen can both cause breast cancer. Hormone replacement therapy (HRT; the process in which endogenous estrogen is administered in menopausal females) also increases the risk of breast cancer (Sun et al., 2017). Androgen hormone is converted to estrogen through an enzyme complex, aromatase. Aromatase is detected in the stromal cell component of the breast; it is also located in the breast epithelial cells. Studies have shown that the level of aromatase was higher in breast tumor cells than in normal cells (Brueggemeier et al., 2003; Pasqualini and Chetrite, 2005). Leptin is another hormone involved in breast cancer; its overexpression causes an increase in cell proliferation and thus leads to breast cancer (Jardé et al., 2011).

Breast cancer is a serious problem that needs to be solved. For this purpose, considerable advances have been made in breast cancer treatment (Howell et al., 2014). Till now, many drugs have been synthesized to cure this deadly disease. Breast cancer-targeted medication utilizes molecules or drugs that suppress breast cancer cell growth in various ways (Maruthanila et al., 2017). Targeted drugs either kill the cancer cells or retard their growth. For example, the expression of abnormal genes such as HER2 (which stimulates breast cancer cell growth) can be blocked by using this medication (Masoud and Pagès, 2017; Lakshmithendral et al., 2019b).

The most commonly targeted breast cancer cell line is MCF-7 because it has been proven to be the most suitable cell line for the investigation of breast cancer all over the world (Lee et al., 2015). MCF-7 cells are universally used for experiments on ER (estrogen receptor) positive breast cancer cells. They are cultured easily, and they maintain their ER expression during treatment with a targeted drug. For this reason, they are highly suitable for anti-hormone therapy resistance studies. MCF-7 cells are very well distinguished and an excellent experience of this cell line permits researchers to utilize these cells to bring more insights into the treatment of breast cancer through viable *in vitro* assays (Comşa et al., 2015).

The use of machine learning has created a revolutionary impact on chemical sciences by quickening the use of computational chemistry methods (Keith et al., 2021). Computer-aided drug designing aims at the discovery and analysis of suitable medications and biologically active compounds by computational approaches. In structure-based drug designing (SBDD), 3D structural information of proteins is utilized to design new drugs by identifying the sites and their interactions that are useful for the biological activity of ligands. In ligand-based drug designing (LBDD), ligand information is utilized to set up an interrelation between their physiochemical characteristics and biological activities. This information is useful for designing new drugs and for the optimization of already known drugs to enhance their activity.

Drug discovery is a costly procedure and time-consuming process; therefore, we have employed computational processes for drug discovery. The advancements in computational methods and high-throughput virtual screening have developed a remarkable pharmaceutical approach that does not only reduce the time phase but also introduces highly efficient drugs, having efficient biological activity and minimum side effects for a specific disease (Lakshmithendral et al., 2019b).

**FIGURE 2:**
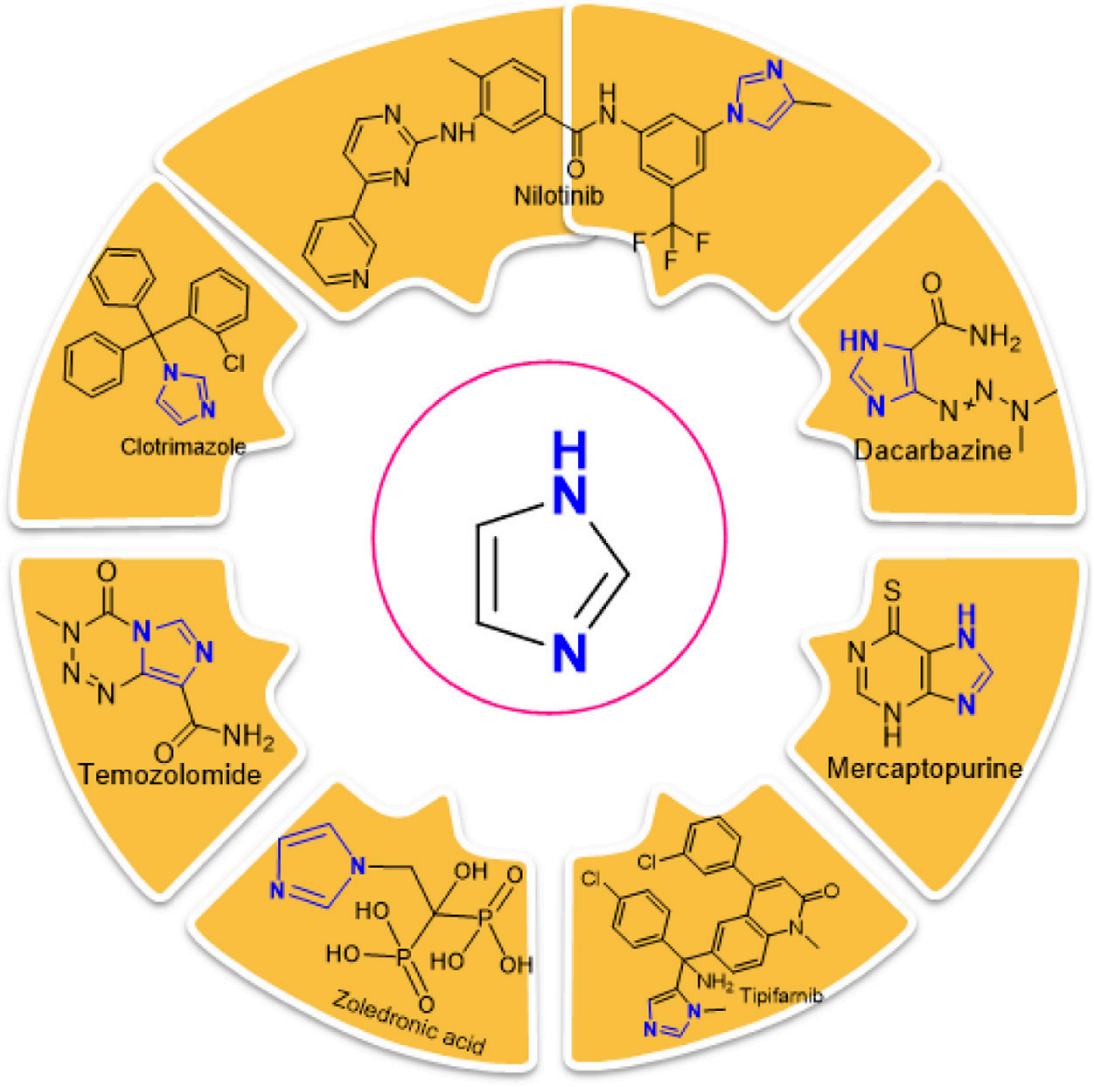
FDA-Approved imidazole derivatives.

Imidazole is the core of FDA-approved drugs with acceptable activities in practice. Several compounds having imidazole core have been utilized for their medicinal uses in clinical trials for several diseases. There is an increasing trend towards imidazole-based medicinal chemistry which has added promising and potential therapeutic values of imidazole-derived compounds for treating incurable diseases. The compounds with imidazole scaffold provide electronic-rich characteristics responsible for binding with a variety of enzymes, proteins, and receptors compared to the other heterocyclic rings. In this study, the role of imidazole drugs as anti-breast cancer agents have been discussed using the computational approach (Chopra and Sahu, 2019). Heterocyclic compounds are very well-known molecules in organic chemistry because they show remarkable medicinal properties as well as anticancer properties (Ali et al., 2017). Imidazoles are very important heterocyclic compounds that are widely utilized all over the world for drug discovery processes and are compounds of interest for researchers for centuries (Gaba and Mohan, 2016). Previous studies have proved the vital role of imidazole and its derivatives in medicinal chemistry because of their efficient uses as anti-coagulant, anti-cancer, anti-parasitic, anti-helmintic, anti-fungal, antimicrobial, anti-inflammatory, antibacterial, anti-viral, anti-diabetic, anti-malarial, antihypertensive, and anti-tubercular drugs (Abbasov et al., 2012; Verma et al., 2013; Mumtaz et al., 2016; Ali et al., 2017). Some FDA-approved anticancer imidazole derivatives are shown in Figure 2.

In spite of extensive studies on imidazole derivatives and their *in vitro* potential activity, their *in-vivo* and, specifically, *in silico* activity of breast cancer has not been carried out. The computer-based methodologies, i.e., *in silico* approaches are powerful tools for the recognition of synthetic imidazole compounds and their potential to inhibit breast cancer. Using these approaches, the new drug candidates can be evaluated in a faster way, reducing costs and accelerating drug discovery (Gowtham et al., 2023). This study aimed to investigate the *in silico* anticancer activities of imidazole derivatives (Rizzo et al., 2014).

In this study, maximum tools used for structure- and ligand-based drug designing have been used, and key regularity features governing the toxicity and anticancer activity of imidazole derivatives have been studied. By discovering and characterizing potential imidazole derivatives as anti-breast cancer agents, this research will contribute to the growing repertoire of drug candidates, expanding the possibilities for future therapeutic interventions. It has the potential to revolutionize the therapeutic landscape by providing new and effective options for breast cancer patients, deepening our understanding of the disease, and inspiring further advancements in the field. It will provide some more valuable insights into the virtual screening and drug designing process and will demonstrate the drug designing process which will lead to drug discovery containing a pharmacophore against breast cancer which is a harmful disease affecting millions of lives all over the world (Alam and Khan, 2017).

## Materials and methods

In this study, the 3D-QSAR modeling has been accomplished using the Forge V6.0 software. A total of 84 compounds (Supplementary Table S1) with reported anti-breast cancer activity were used while developing the model. An FDA-approved drug Fulvestrant which is a steroidal anti-estrogen used to treat hormone-receptor-positive metastatic breast cancer is used as a reference compound.

### The development of the 3D-QSAR model

#### Data collection and structure preparation

The dataset of imidazole compounds was collected from prior reports/literature. Their structures were drawn in Chem Draw professional (Perkin Elmer), and 2-Dimentional structures were converted into 3-Dimentional structures using Chem3D Ultra (Version 19.1.0.8, Perkin Elmer). The value of enzyme inhibition (experimental activity) was expressed as (IC_50_) for the training dataset which was then altered to its positive logarithmic scale using the formula: pIC_50_ = −log(IC_50_) and defined as a dependent variable. The database of compounds was generated in Microsoft Excel as a CSV output file (comma delimited).

#### Conformation hunt and pharmacophore generation

To demonstrate a hypothesis for 3D conformation, the Field Template module of Forge V6.0 software was used as no structural data was attainable for imidazole derivatives in their target-bound state. For this purpose, the information about field and shape was utilized by the template from the library of 84 compounds. The hypothesis was developed by generating the three-dimensional field point pattern and calculating the field points of bioactive conformation.

#### Compound alignment and the development of the 3D-QSAR model

At the connexion point of a 3D grid, the 3D-QSAR method calculates various molecular properties as molecular descriptors. This methodology covers the complete data of aligned training set compounds. The pharmacophore template was transported into the Forge V6.0 software, followed by the alignment of compounds with the associated template. After the alignment of 84 compounds with known IC_50_ values, the 3D-QSAR model was built using the Field point-based descriptors. While building the model, the maximum distance of sample points was set to 1.0Å, the maximal number of components was set to 20, Y scrambles were adjusted to 50, and volumetric as well as electrostatic fields were also used. For overall resemblance, 50% dice volume similarity and 50% field similarity were achieved using the Forge software. The experimental activity (IC_50_) of compounds was changed to pIC_50_ which is equal to the negative log of IC_50_. The set of 84 compounds was divided into the training set and test set with a ratio of 80% and 20%, respectively, and one compound was selected as the reference drug to assess the QSAR modeling using the activity stratified method.

#### QSAR model validation

The model was verified by q^2^ (cross regression coefficient), *r* ^2^ (regression coefficient), and similarity score of conformers for every ligand. LOO technique (leave-one-out) was used to assess the derived 3D-QSAR model. The LOO cross-validation technique is thought to be one of the most efficient techniques for the validation of the regression model having a small training dataset. The data size of N-1 was used for training and the remaining one was tested; N identifies the complete dataset. In the LOOCV technique, the process of testing and training was repeated for the N number of times, and in this way, each data was passed through the testing method. Then, the test data which is not in the training set is used to derive the 3D-QSAR model.

#### SAR activity-atlas models visualization

The global aspect of training data was studied quantitatively by using the Bayesian approach. The hydrophobicity, electrostatics, and shape attributes, which lie beneath the SAR of a particular set of compounds, are better understood by this approach. These 3D models were viewed to achieve valuable information. The three types of interconnected biochemical evaluated data including regions explored analysis, activity cliff summary, and average of actives were revealed by the Activity-atlas study. The regions explored analysis exhibited the areas of aligned and fully explored compounds. The details about negative and positive electrostatic sites, appropriate and inappropriate hydrophobicity, and appropriate shape of actives were provided by the activity cliff summary. On the other hand, an average of actives helped in showing the common parts in active compounds which were selected.

### Target prediction analysis by molecular docking

#### The preparation of protein

**FIGURE 3:**
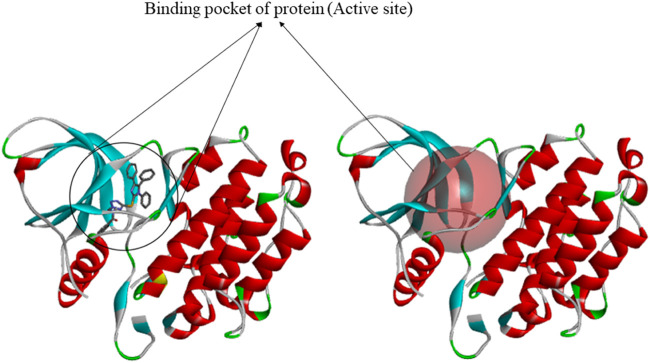
Generation of binding site by using discovery studio.

The 3D structures of target proteins (*PDB ID: 7CHM, 3PP0, 4UDD, 5NWH, 3HY3, and 4ZVM*) **(** Supplementary Table S2 **)** were downloaded from the RCSB PDB database (https://www.rcsb.org/). The protein preparation was performed to accomplish various tasks such as identifying the active site, deleting alternate conformations, interpolating missing atoms in incomplete residues, protonating titratable residues, modeling the missing loop areas, and removing the water molecules and heteroatoms (Alam and Khan, 2019). The ligands of proteins were used to identify the active sites from the “Define and Edit Binding Site” option in Discovery Studio, and SBD_Site_Sphere was generated (Figure 3).

#### The preparation of active ligands

Active ligands along with reference Fulvestrant (standard drug) having known inhibitory potentials were collected from literature, and SDF files of some of the compounds were downloaded from PubChem while others were generated from the Chem3D software where they were optimized through MM2 and MMF9 force field. For ligand preparation, the “Open Babel” software was used. For this purpose, the input file was imported in the form of sdf–MDL MOL format and the output path was selected in pdb format. Then the ligand was converted into the desired form.

#### Protein-ligand docking studies

PDBQT files of proteins and ligands were prepared, and by using the Graphical User Interface program AutoDock Tools, grid box creation was accomplished. Fragmental volumes to the protein, polar hydrogens, united atom Kollman charges, and solvation parameters were assigned by AutoDock tools. After preparation, all files were saved as PDBQT. Preparation of the grid map was done by using a grid box via AutoGrid. The grid size was selected as 60 × 60 × 60 XYZ points with a grid spacing of 0.375Å. Default settings were used for all other parameters. Autodock was used for docking protocol and information about proteins and ligands was used along with grid box features in the configuration file. Both ligands and proteins are considered rigid when using Autodock. The results lower than 1.0Å in the root-mean-square deviation were assembled and were depicted by the result with the most suitable free-binding energy while the results with the lowest binding affinity or binding energy were extracted and subjected to further analysis (Azam and Abbasi, 2013).

### Molecular dynamics simulation

MD simulation was accomplished via the iMOD server (https://imods.iqfr.csic.es/) to assess the physical movement and stability of protein-ligand complexes (Sumera et al., 2022). The structural dynamics of the protein-ligand complexes were analyzed using iMODS and the molecular motion was also determined. The iMOD server employs Normal mode analysis (NMA) to calculate the internal coordinates of protein to evaluate its stability. In this study, the conformational fluctuations of docked complexes were demonstrated and their slow dynamics were investigated using NMA (Kirar et al., 2022).

### Toxicity prediction

The toxicity of the compounds was determined using ProTox-11 (Banerjee et al., 2018). With the help of this tool, the toxicity of compounds can be freely estimated by inserting the name of the compound or by simply writing its canonical smiles. The 2-Dimensional structure of the compound is used as input for this webserver. ProTox-11 is distributed in different classes depending on the toxicity such as Organ toxicity, immunotoxicity, carcinogenicity, cytotoxicity, and mutagenicity.

### Geometry optimization and reactivity determination

The DFT calculations were performed using Gauss view 06 and Gaussian. The 2D structure of the molecule was drawn by using Perkin-Elmer ChemDraw and then converted into a 3D structure through the Chem3D software. Geometrical optimization was done with B3LYP (an exchange-correlation function) and basic sets of 6-311G (Türker et al., 2010). The reactivity and stability of the compound were determined by calculating the energy gap between the HOMO-LUMO orbitals.

## Results and discussion

### 3D-QSAR modeling on imidazole derivatives

#### Conformation hunt and pharmacophore generation

**FIGURE 4:**
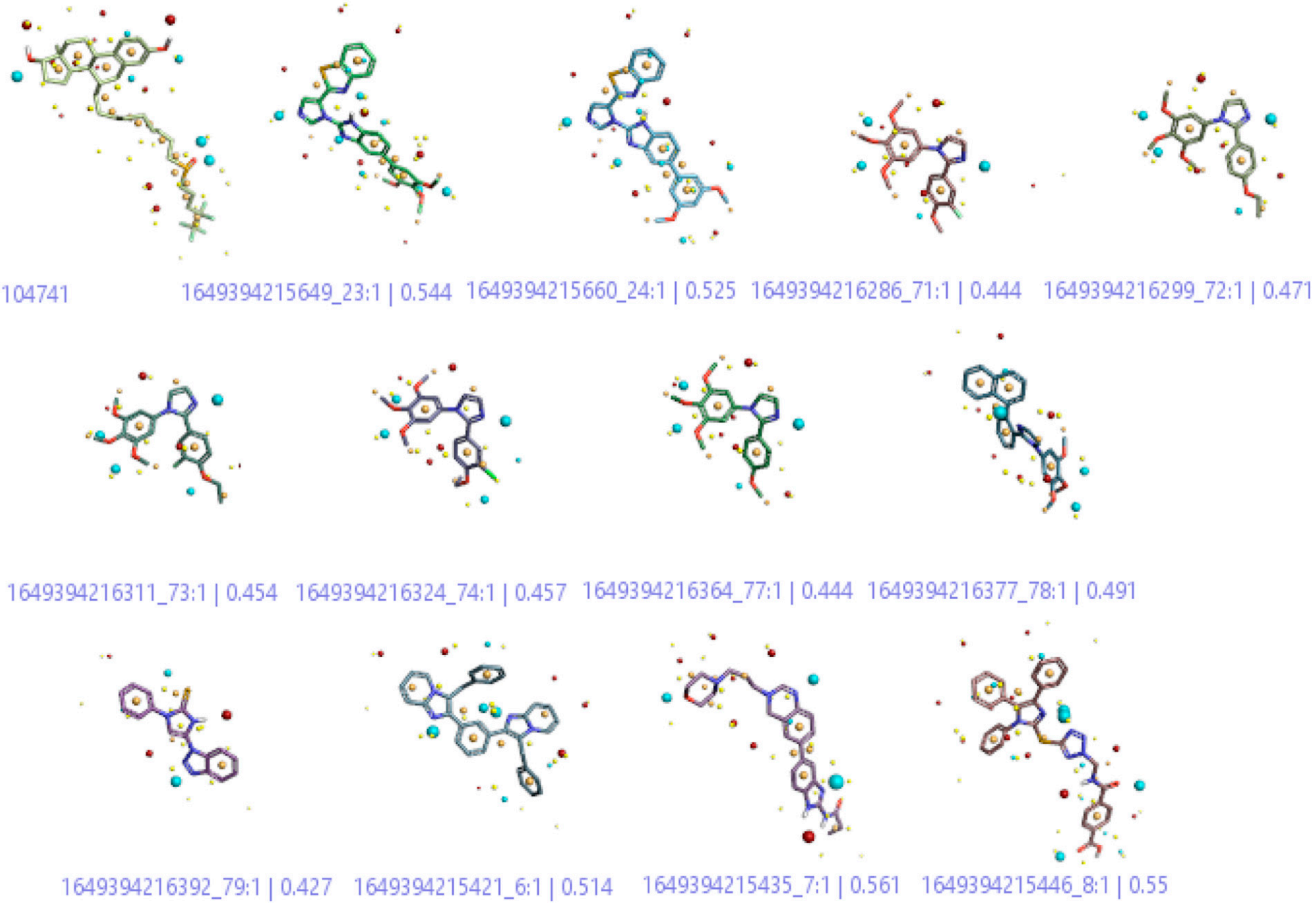
3-Dimenssional Field points for QSAR model development. The red colour indicates positive electrostatic potential while negative electrostatic potential is represented by the blue colour. The orange color shows hydrophobicity and the yellow color denotes van der Waals descriptors localization.

A three-dimensional structure-activity relationship (3D-QSAR) was performed to throw more light on a series of imidazoles. For this purpose, a conformational hunt was carried out on these compounds (**C1-C84**). (Alam and Khan, 2017). The three-dimensional pattern of field points (Figure 4) was identified by illustrating the derived conception of bioactive conformation with its calculated field points. Four distinct molecular fields were calculated, namely, negative and positive electrostatic potential, hydrophobicity, and shape/van der Waal descriptors. To draw a pharmacophore template resembling the bioactive conformation (for further virtual screening), a molecular field-based similarity approach was employed.

Figure 4 shows that the compounds with similar filed points bind at the same target site. This characteristic provides a linear correlation between biological activity and structural similarity of ligands (Low et al., 2005). Based on the parameter, Figure 4 shows the active ligands, and their similarity metric was found within the range of 56% to 42%.

#### Alignment and development of the 3D-QSAR model

**FIGURE 5:**
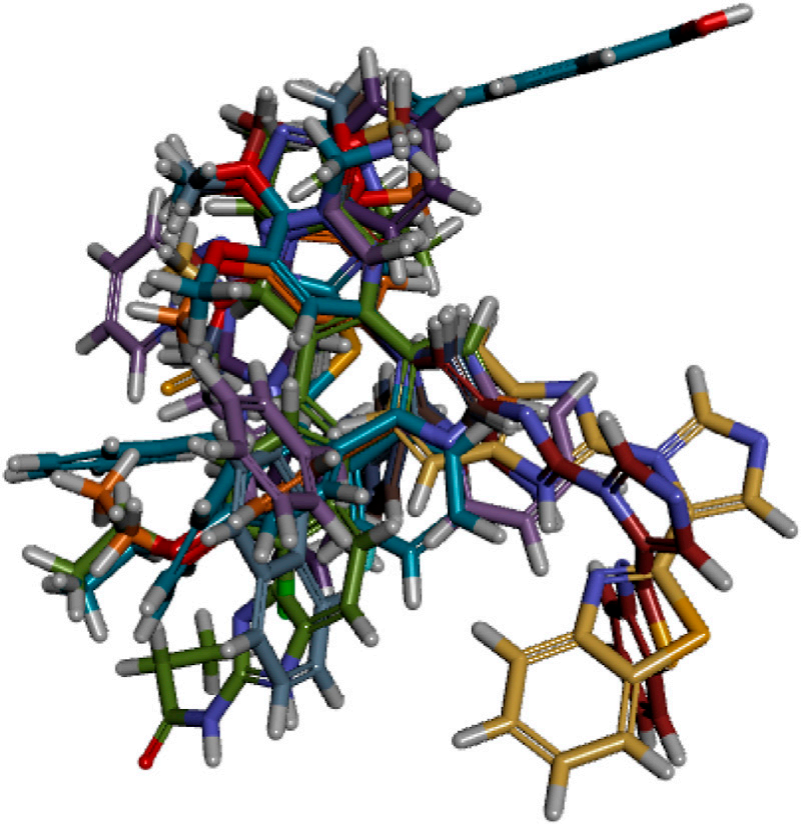
Conformational alignment of active compounds.

The ligand alignment in the protein context is required to use the 3D similarity metric for activity atlas model development. To ensure accurate model development, this alignment must be inspected. The compounds in the training set were aligned to ensure that the molecules being compared were in the same relative orientation (Figure 5). This alignment is necessary because molecules can adopt different conformations or spatial arrangements due to the freedom of rotation around single bonds. After it, the 3D-QSAR model was built by using the Field points-based descriptors. The activity interactive graph plot was used to represent the fitness of the derived 3D-QSAR model. This graph displays the comparison between predicted and actual activity with cross-validation data points. Fairly good activity-descriptors’ relationship accuracy of 81% was achieved by the derived 3D-QSAR model as the regression coefficient was *r* ^2^ = 0.81. Similarly, as mentioned by the cross-validation regression coefficient (q^2^ = 0.51), a high activity-prediction accuracy of 51% was attained. The derived 3D-QSAR model was proved to be very reliable to predict the anticancer and cytotoxic activity of imidazole derivatives as an MCF7 cell-line inhibitor (Table 1).

**TABLE 1:** Active compounds obtained after the 3D-QSAR model development.

Sr No	Compounds	Structure	IC_50_ (µM)	PIC_50_	References
**1**	**C6**	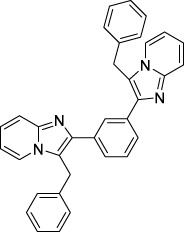	0.30	6.5228	Meenakshisundaram et al. (2019)
**2**	**C7**	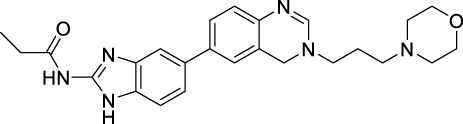	0.38 ± 0.08	6.4202	Fan et al. (2020)
**3**	**C10**	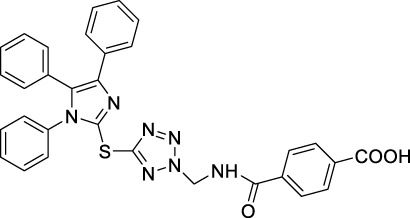	0.38 ± 0.04	6.4202	Al-Blewi et al. (2021)
**4**	**C21**	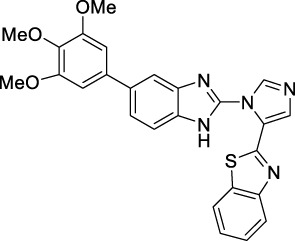	0.018 ± 0.0039	7.7447	Edukondalu et al. (2021)
**5**	**C22**	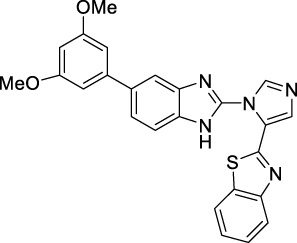	0.10 ± 0.028	7	Edukondalu et al. (2021)
**6**	**C68**	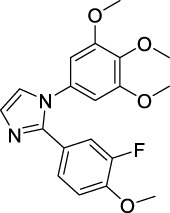	0.074 ± 0.017	7.1307	Romagnoli et al. (2016)
**7**	**C69**	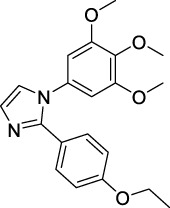	0.0015	8.8239	Romagnoli et al. (2016)
**8**	**C70**	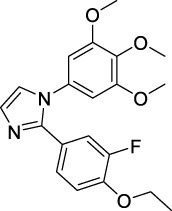	0.0034	8.4685	Romagnoli et al. (2016)
**9**	**C71**	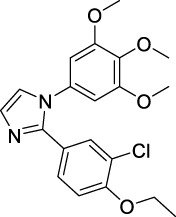	0.0007	9.1549	Romagnoli et al. (2016)
**10**	**C74**	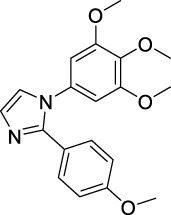	0.23	6.6382	Romagnoli et al. (2016)
**11**	**C75**	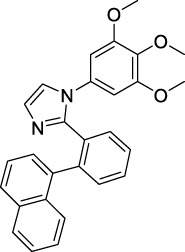	0.0046	8.3372	Romagnoli et al. (2016)
**12**	**C76**	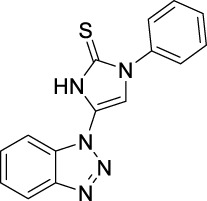	6.4 ± 0.18	8.1938	Khayyat et al. (2021)

Figure 5 shows that there were displayed little spaces in ligands to accommodate small conformation changes and variations of moieties present in aligned molecules. High molecules have tight alignment to restrict the substitution or replacement of any group present in them. Low-active molecules, in comparison, lack steric tightness and have the capacity to substitute any moiety in the context of an activity enhancer (Low et al., 2005).

### The regulation of the SAR mechanism of imidazole derivatives by field points

#### The identification of field points (coefficient and variance) governing the anticancer activity

**FIGURE 6:**
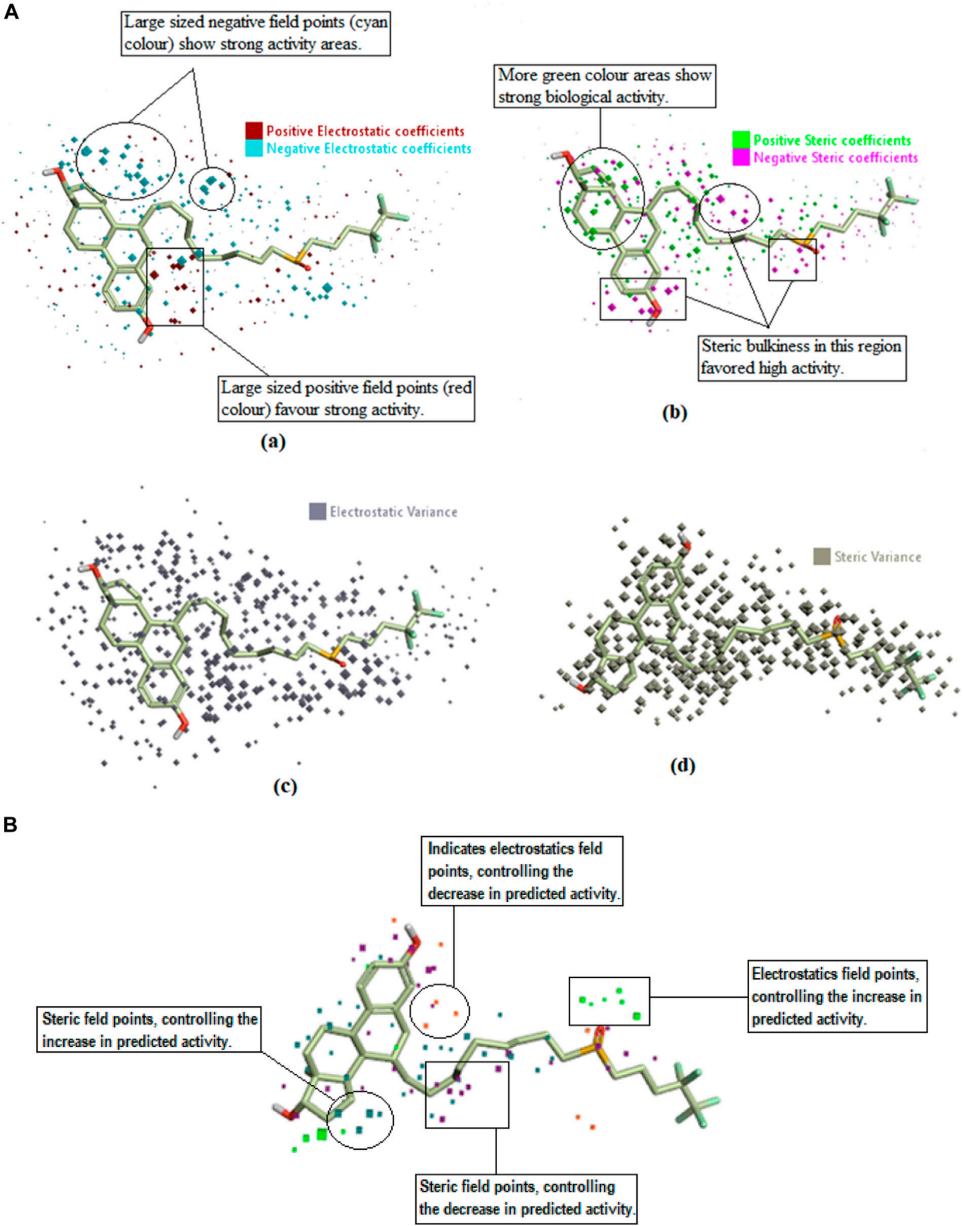
**(A)** Coefficients and variance field points of Fulvestrant; (a) Electrostatic coefficient; (b) Electrostatic variance; (c) Steric coefficients; (d) Steric variance; (e) Field contributions to the predicted activity. **(B)** Field contributions to the predicted activity.

The QSAR model was also viewed in a 3D form to unveil the structure-activity relationship (SAR) mechanism of imidazole derivatives. The field points named coefficient and variance (associated with the bioactivity of training set compounds) were analyzed in a 3D structural form for the purpose. The derived model points for QSAR were contrasted with the reference compound for better comprehension of space field point localization (Alam and Khan, 2014). In a robust model, the high coefficient and variance field points were proved to be the highly essential correlating parameters. According to the results, electrostatic and steric coefficients both play a major role in modulating the anticancer activity as represented by the large size of red, cyan, green, and pink field points (Figure 6). Field points containing high steric and electrostatic variance indicated regions of high changes while the field points containing low variance represented the regions with less changes or no changes (Figure 6).

#### Field contribution in activity prediction

“View field contributions to predicted activity” study was done on imidazole derivatives. This evaluates the extent to which imidazole derivatives fit the derived field-based 3D-QSAR model and regions of structural field points governing the predicted activity. These field contributions (Figures 6A, B) were represented by purple, blue, and red color regions. According to the results, the orange- and purple-colored areas denote the region of electrostatics and steric field points, respectively, having the negative regulation capability on predicted activity (decrease anticancer activity). Whereas the green- and zinc-colored areas denote the regions of electrostatics and steric field points, respectively, with a positive regulation capability on the predicted activity (increased anticancer activity).

#### Activity-atlas visualization for SAR mechanism identification

SAR study was practiced through the activity-atlas visualization technique and was used to unveil the key features of imidazole, regulating the anticancer activity and designing more novel drugs. For this purpose, an activity cliffs summary and an average of actives study were performed on imidazole derivatives.

#### Average of actives model

**FIGURE 7:**
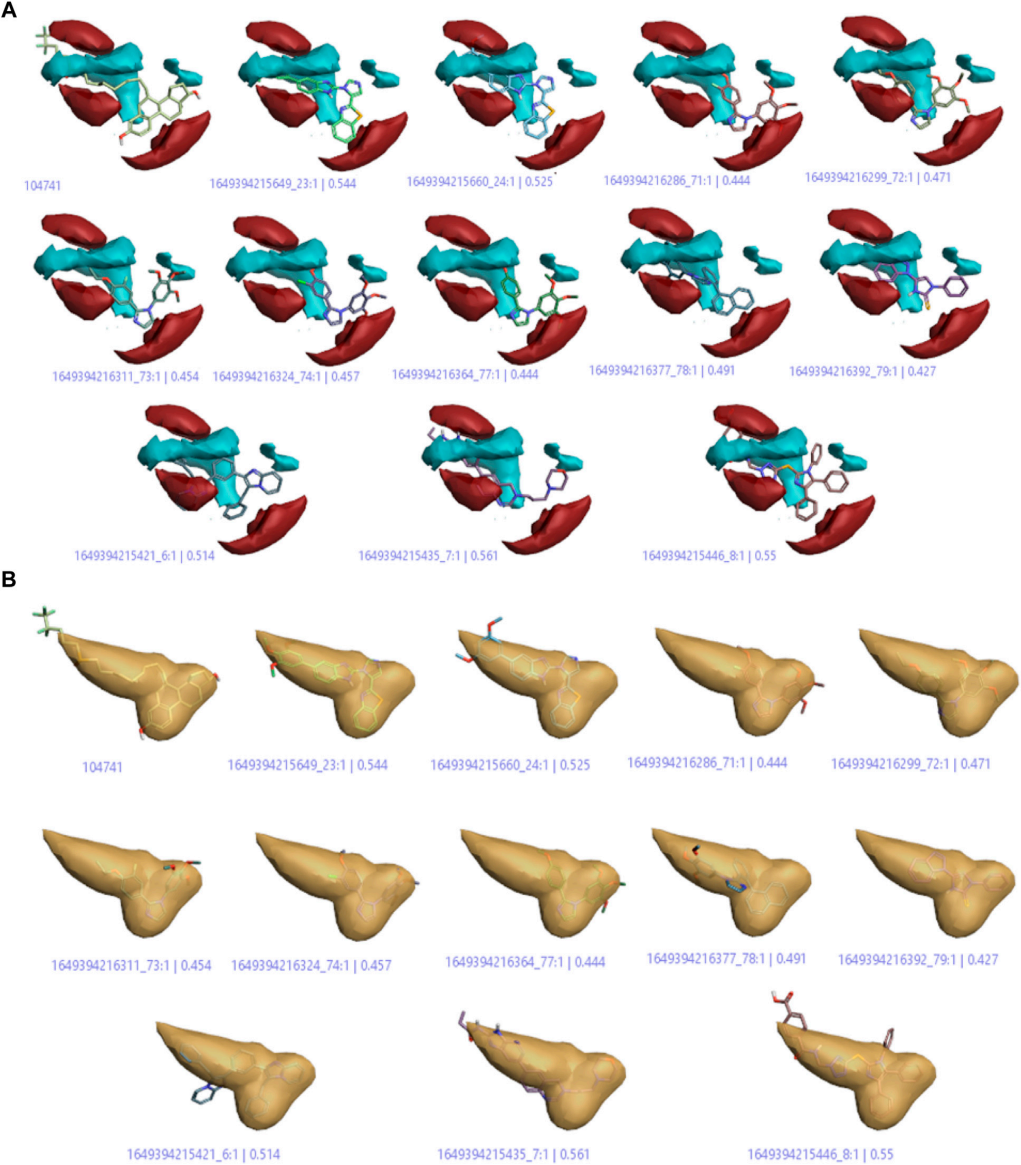
**(A)** Positive and negative electrostatic regions **(B)** The hydrophobic interaction regions of active compounds.

On the basis of this model, imidazole compounds having a pIC_50_ value higher than 6.4 were classified as active compounds while the rest of the compounds were considered inactive. This model (Figure 7; Fig. S1) represents the areas of high activity that reference drugs and active ligands have in common.

In Figure 7A, the positive and negative electrostatic regions represented by the red color sites and cyan color sites, respectively, correlate with the anticancer activity, i.e., more of these regions indicate more anticancer activity. Figure 7B shows the hydrophobic interaction regions of active compounds as indicated by the yellow color, and Supplementary Figure S1 shows the shape regions of active compounds as represented by the white color.

#### Activity cliff summary

**FIGURE 8:**
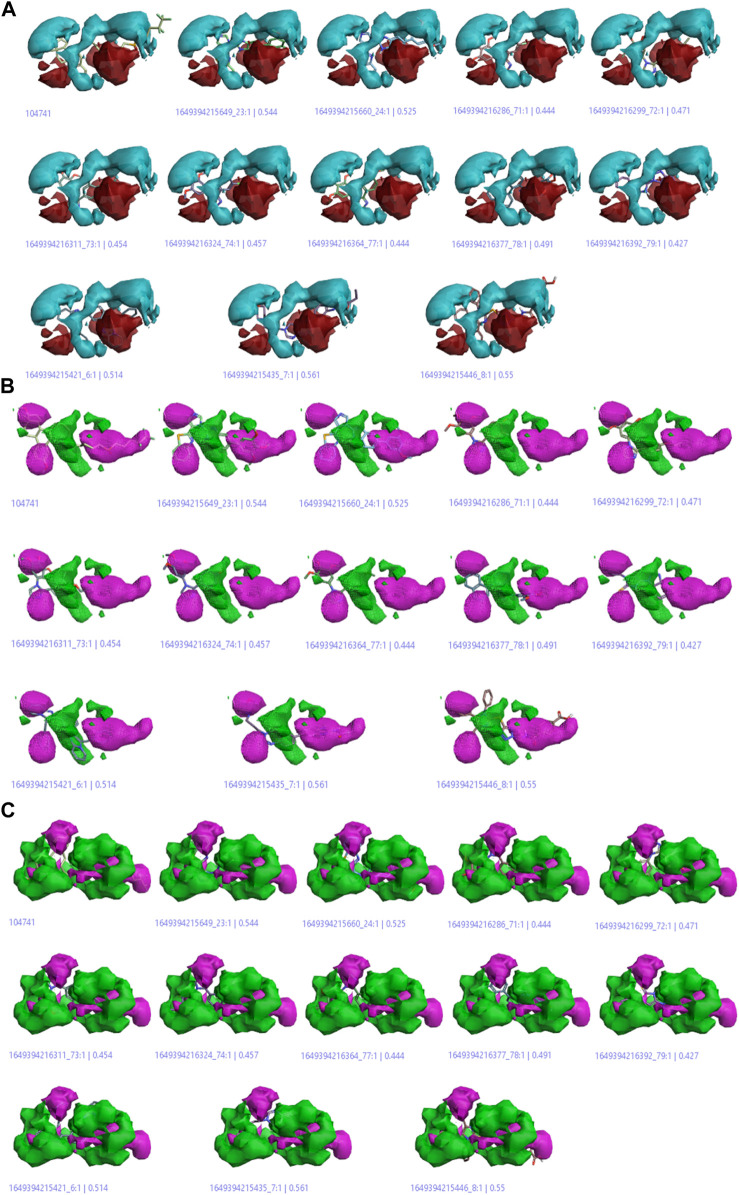
**(A)** Cliff summary of electrostatics. **(B)** The areas of favorable and unfavorable hydrophobics. **(C)** Favourable shape region and unfavorable shape region.

The activity cliff summary diagrams as indicated by Figure 8A correlate with the biologically active parts of imidazole drugs with the reference drug. The cliff summary of electrostatics in Figure 8A is visualized in two colors: red and cyan. The presence of the red color indicating the positive electrostatic field and the cyan color indicating the negative electrostatic field is favorable for high anticancer activity. Figure 8B shows the areas of favorable and unfavorable hydrophobics represented by the green and purple colors, respectively. Whereas in Figure 8C, the green color shows a favorable shape region, and the purple color shows an unfavorable shape region.

#### Regions explored

**FIGURE 9:**
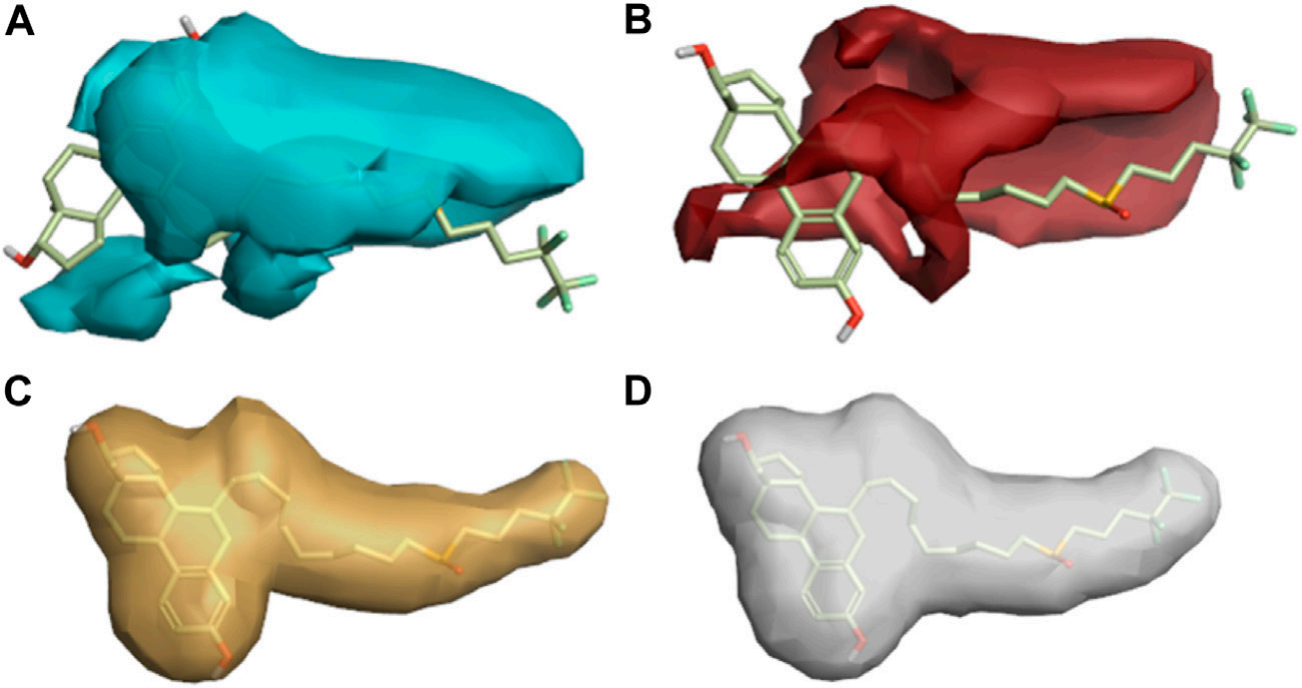
Regions explored for Fulvestrant by active molecules in the activity atlas model. **(A)** Regions explored in negative electrostatics, **(B)** Regions explored in positive electrostatics, **(C)** Regions explored in Hydrophobic, and **(D)** Shape explored.

The descriptive features of compounds were explored in this model aside from their biological activities (Attiq et al., 2022). The more red and cyan colors indicating positive and negative fields, respectively, show the areas of strong SAR with the reference drug. The average regions explored also represent the areas of active compounds that would not take part in an anticancer activity (Figure 9).

#### Validation of the 3D-QSAR model

Molecular characteristics regulating the active compounds as anticancer agents were retrieved for further prediction of their anticancer activity based on derived SAR models. Before that, prediction performance was analyzed on the test set and training set compounds by predicting their anticancer activity. This prediction was done by means of derived models and then the distance value (error) was compared. In order to perform the comparison, predicted activity and distance to models’ columns were examined for each derived model. The important ligand fields were illustrated for each derived model through this study and after that, these characteristics were utilized for virtual screening.

### Ligand-based virtual screening

To predict hits, a series of ligand-based virtual screening experiments were performed. Only high-hit compounds were selected having the value of ‘excellence’. The excellence of hits was set by taking a threshold of docking score −8.7 kcal/mol to compare the biological activity. The predicted activities were expected to be reliable because most of the characteristics in compounds were the same as the training set. In contrast, compounds having poor field point similarities were excluded to evade the false positive compounds by ineffective predicted activities. Also, the derived QSAR model was used to predict the hit compounds for anticancer activity (Lakshmithendral et al., 2019b).

#### Structure-based virtual screening (SBVS)

SBVS of selected compounds was performed to discover new valuable drugs in order to treat breast cancer (El Aissouq et al., 2021). AutoDock Tools provided notable results with overall binding energy of all the selected compounds ranging from −5.1 to −10.5 kcal/mol. Most of the compounds have binding free energy greater than the reference compound Fulvestrant when observed with all the selected proteins as shown in Table 2.

**TABLE 2:** Binding energy values in kcal/mol.

	Proteins PDB IDs					
Compounds	4ZVM	5NWH	7CHM	3HY3	4UUD	3PP0
**C6**	−10.3	−8.7	−9.4	−8.1	−8.8	−7.6
**C7**	−7.2	−6.2	−8.6	−8.2	−6.4	−10.0
**C10**	−7.9	−7.8	−10.5	−9.0	−8.0	−8.1
**C21**	−8.3	−7.8	−9.2	−9.7	−9.1	−7.2
**C22**	−8.9	−7.5	−9.2	−8.7	−8.8	−9.0
**C68**	−6.6	−5.9	−8.3	−6.6	−5.4	−8.3
**C69**	−6.2	−6.1	−7.6	−6.7	−5.1	−7.6
**C70**	−6.6	−5.8	−8.1	−6.9	−5.2	−8.1
**C71**	−6.1	−5.9	−7.5	−6.1	−5.1	−7.5
**C74**	−6.1	−6.1	−7.6	−6.4	−5.3	−7.6
**C75**	−6.2	−6.6	−8.3	−6.1	−6.5	−8.3
**C76**	−7.5	−6.9	−8.9	−8.5	−6.2	−10.3
**References**	−6.8	−6.2	−7.9	−8.2	−6.1	−6.6

The docking results explain that compounds **C6, C10, C21,** and **C76** are the most hit compounds giving excellent results, so only these compounds will be subjected to further study.

Molecular Interaction and Binding Mode. The top hit compounds represented by the shaded area in Table 2 were selected to evaluate the binding site interactions between the ligand and the target protein.

**FIGURE 10:**
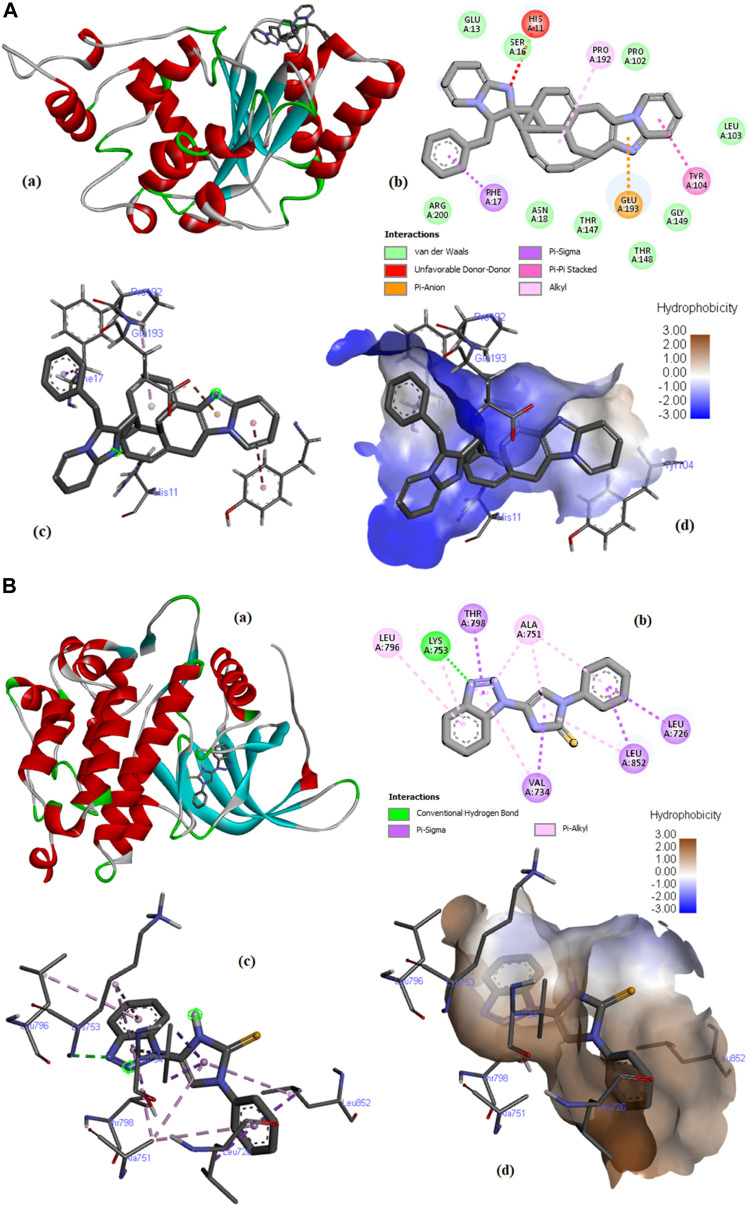
**(A)** Molecular docking of compound **C6** with PDB ID: 4ZMV; (a) 3D view of the best-selected conformation, (b) 2D Interactions, (c) Ligand interactions, and (d) Hydrophobicity. **(B)** Molecular docking of compound **C76** with PDB ID: 3PP0 (a) 3D view of the best-selected conformation. (b) 2D Interactions. (c) Ligand interactions. (d) Hydrophobicity.

Compound **C6** was fixed in the binding pocket of protein **(PDB ID: 4ZVM)** (Figure 10A) by undergoing electrostatic interactions (Pi-Anion) with GLU193 and hydrophobic interactions (Pi-Sigma, Pi-Pi Stacked, and Alkyl) with PHE17, TYR104, and PRO192 (Supplementary Table S3).

The docking of compound **C6** with protein **(PDB ID: 5NWH)** is described in Supplementary Figure S2. **C6** undergoes interactions with the protein by hydrophobic interactions (Pi-Sigma and Pi-Alkyl) with VAL 49, VAL29, and PRO86, Pi-Cation electrostatic interactions with ARG196, and other interactions (Pi-Sulfur) with CYS91 (Supplementary Table S3).

Supplementary Figure S3 describes the docking of compound **C10** with the protein having PDB ID: 7CHM. The compound fits in the binding pocket of the protein through hydrogen bonding (Conventional Hydrogen Bond and Carbon Hydrogen Bond) with amino acids ASP608, SER611, GLN530, ASP674, and ILE607; hydrophobic interactions (Pi-Sigma, Pi-Pi Stacked, Alkyl, and Pi-Alkyl) with ILE531, ILE607, LEU654, ALA651, PRO673, VAL539, ILE663, and ALA551; and miscellaneous interactions (Pi-Sulfur) with MET602, CYS604, and MET671 (Supplementary Table S4).

However, the docking of compound **C21** with **PDB ID: 3HY3** provided different results (Supplementary Figure S4). **C10** interacted by forming a conventional hydrogen bond with TRP109 and hydrophobic interactions (Pi-Pi Stacked, Pi-Pi T-shaped, Alkyl, and Pi-Alkyl) with TYR83, TRP109, PRO81, MET90, TYR152, TYR153, and LYS150 (Supplementary Table S4) (Elancheran et al., 2023a).

Compound C**21** fits in the binding pocket of protein (PDB ID: 4UDD) (Supplementary Figure S5) through hydrogen bonding (conventional hydrogen bond and carbon-hydrogen bond) with GLN642, GLN738, and PRO637, hydrophobic interactions (Pi-Pi Stacked) with TRP557 and TYR735, and miscellaneous interactions (Pi-Sulfur) with MET745 (Supplementary Table S2). Docking results of Compound **C76** with **PDB ID: 3PP0** are described in Figure 10B. C76 interacted by forming conventional hydrogen bond with LYS753 and hydrophobic interactions (Pi-Sigma and Pi-Alkyl) with LEU726, VAL734, THR798, LEU852, ALA751, and LYS753 (Supplementary Table S2).

### Validation of docking

**FIGURE 11:**
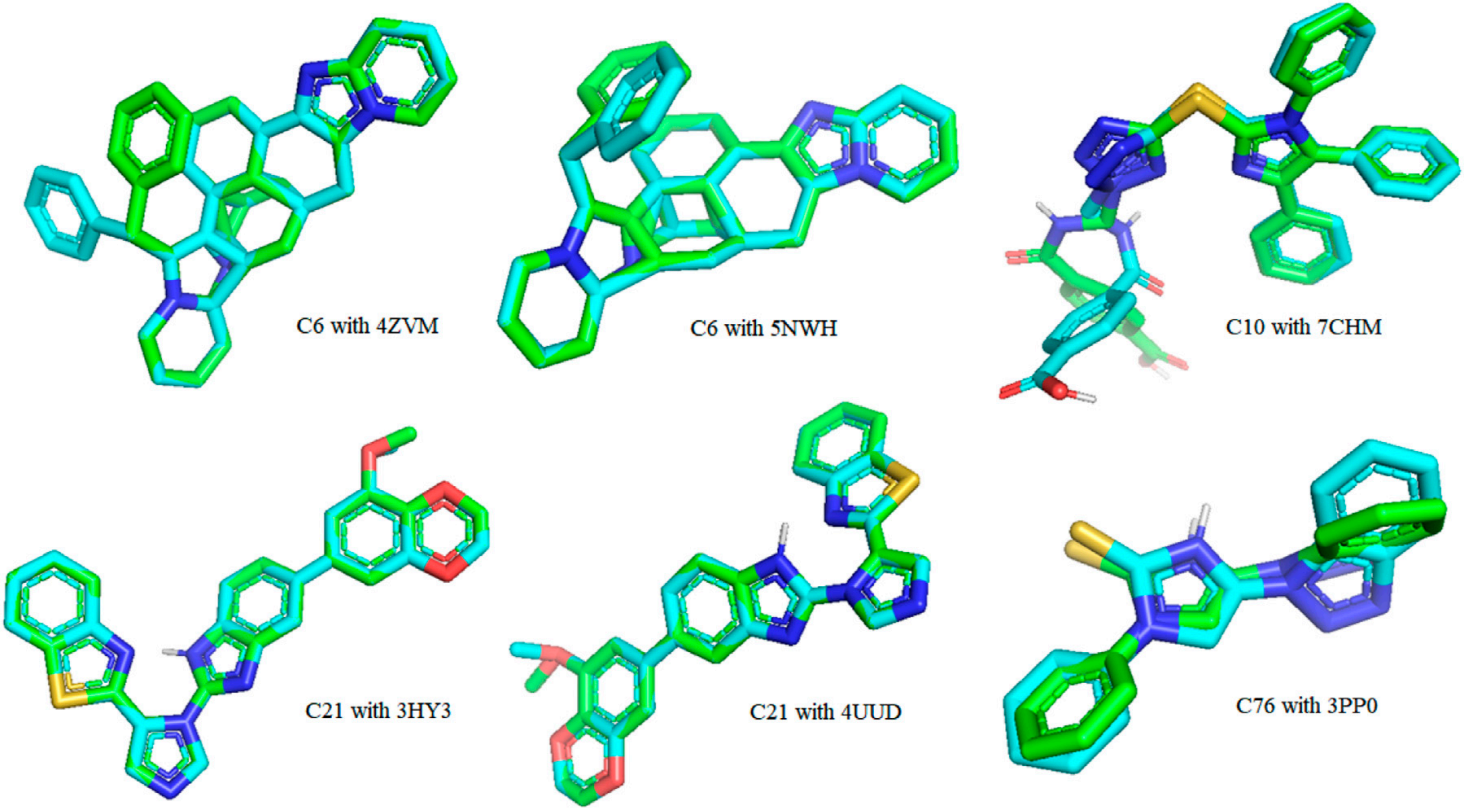
A 3-dimensional view of the best-docked pose of the ligand that fitted into the binding pocket of the protein receptor binding site.

The re-docking of the native ligand with the protein receptor binding site was performed to validate the docking process by using the PyMOL molecules graphic system, version 2.4.1. The crystal structures were aligned to compare their changes in conformation and displacement. The results were reported in root-mean-squared deviation (RMSD) to calculate the deviation between analogous atoms of two proteins, i.e., the docked pose and the corresponding crystal conformer. Redocking of all hit compounds with PDB IDs 4ZVM, 5NWH, 7CHM, 3HY3, 4UUD, and 3PP0 resulted in the RMSD values shown in Supplementary Table S5. The lower value of RMSD revealed that the ligands were bound to target very closely to the original conformation, hence, signifying the accuracy of results. The RMSD value close to zero was considered to be ideal. A superimposed view is displayed in Figure 11 **.**

#### Molecular dynamics simulation

**FIGURE 12:**
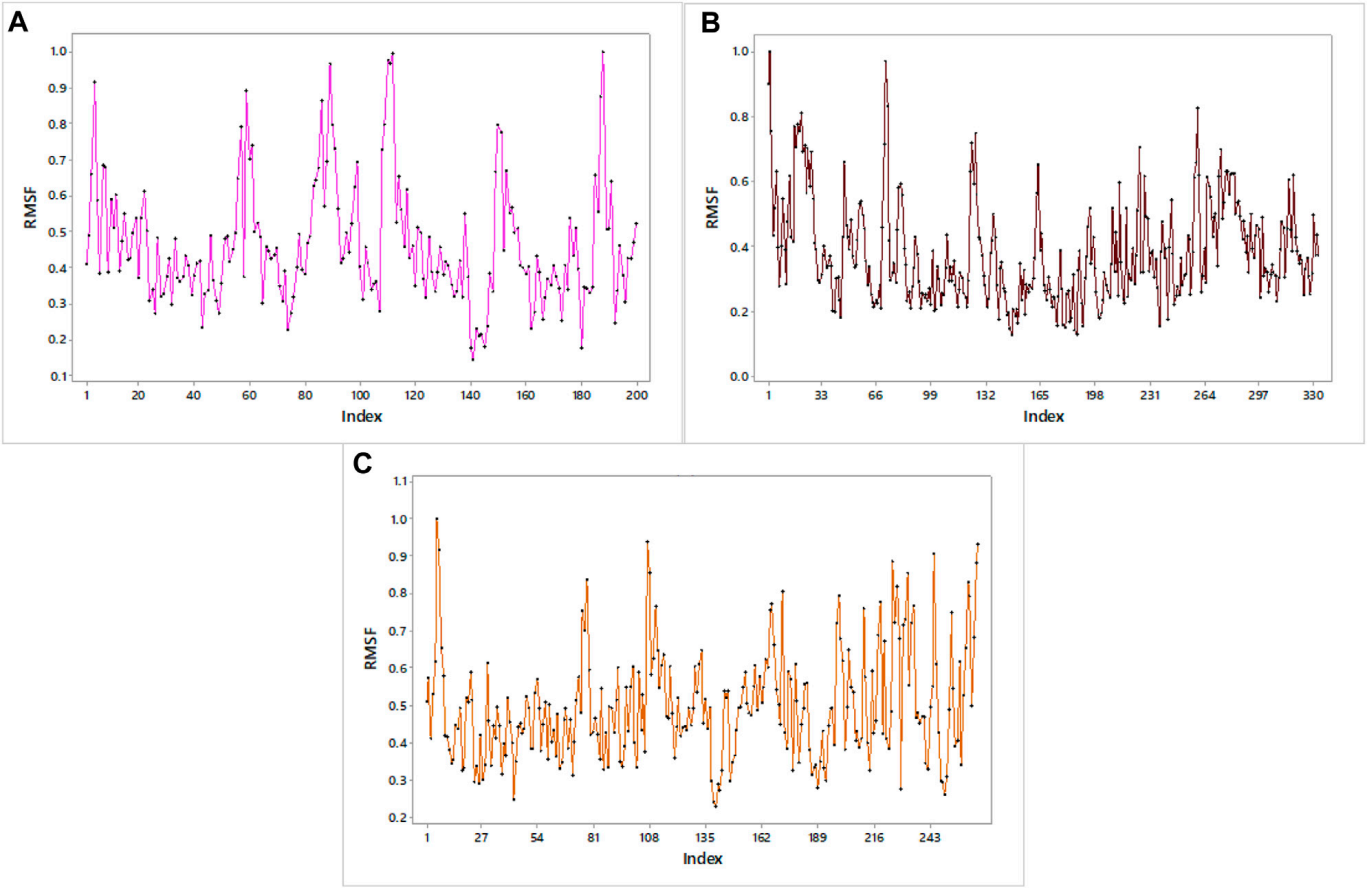
RMSF profiles of **(A)** H3Y3, **(B)** 4UUD, and **(C)** 7CHM.

The RMSF graph provides insight into the flexibility of individual atoms or residues in the protein. It shows how much they deviate from their average positions during the simulation. The maximum value of RMSF indicates greater flexibility, while the smallest value denotes the system’s restricted motion across the simulation course. In all of our proteins, the RMSF graph showed a number of areas having high flexibility (Figure 12). The complex of C10 with H3Y3 showed a maximum number of peaks, indicating more flexible movements. All three docked complexes showed a maximum RMSF of 1.0. It indicated that the atoms or residues are, on average, deviating from their average positions by around 1 Å (Angstrom).

**FIGURE 13:**
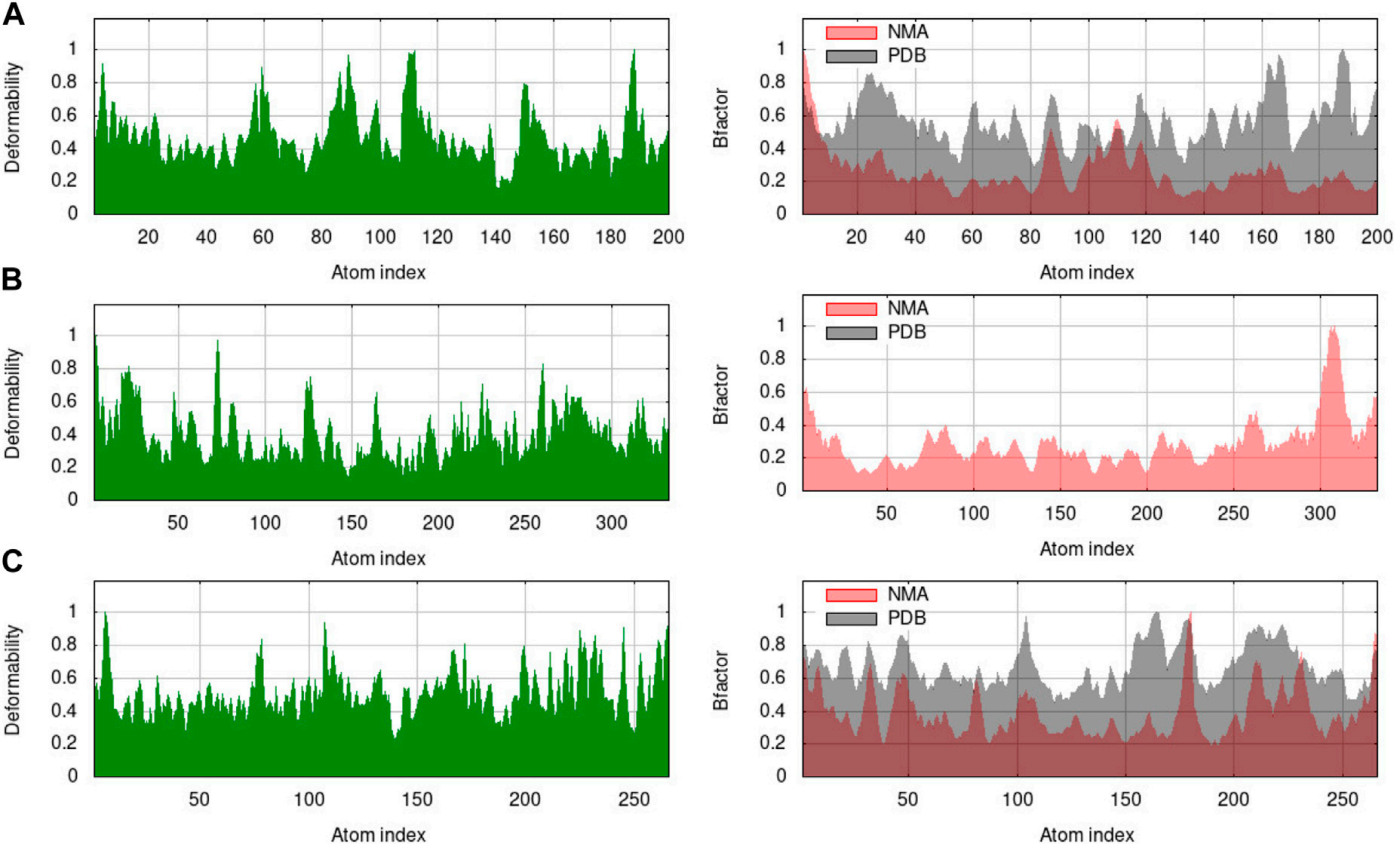
Deformability and B-factor of docked complexes. **(A)** 3HY3-C10, **(B)** 4UUD-C10, and **(C)** 7CHM-C10.

The B-factor, eigenvalue, deformability, covariance matrix, variance map, and elastic network model of the protein serves as a representation of its stability. The mobility characteristics of the docked proteins are determined by the deformability and B-factor. The peaks are associated with the protein regions with deformability whereas the areas with the highest peaks are those with the greatest deformability (Santra and Maiti, 2022). In B-factor graphs, the comparison between the PDB field and NMA of the docked complexes is provided. The B-factor graphs of 3H3Y-C10 and 7CHM-C10 complexes showed that the PDB data predicted higher B-factors compared to the NMA data. It suggested that the B-factor values predicted by the computational simulations using NMA showed lower mobility or flexibility than what was predicted by the experimentally determined B-factor values from the Protein Data Bank. Figure 13 illustrates the deformability and B-factor of 3HY3-C10, 4UUD-C10, and 7CHM-C10, respectively.

**FIGURE 14:**
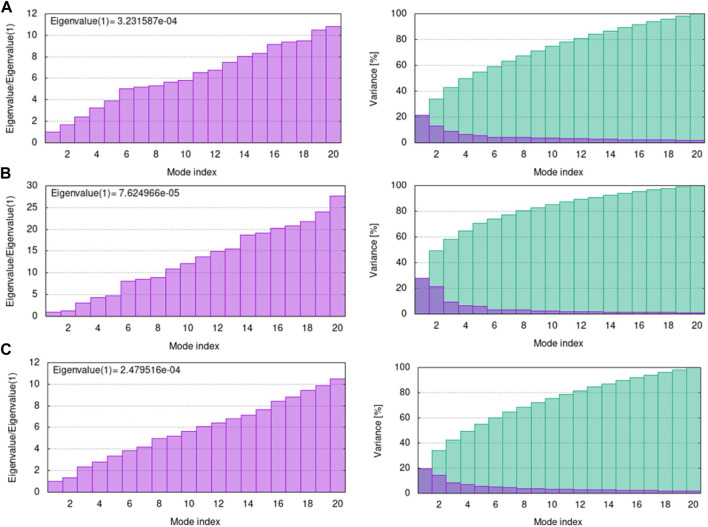
Eigenvalue and variance of docked complexes **(A)** 3HY3-C10, **(B)** 4UUD-C10, and **(C)** 7CHM-C10.

The eigenvalue indicates motion stiffness which is directly connected to the energy needed to deform the structure. If the eigenvalue is low, it means that the complex is more easily deformable. The eigenvalues of 3H3Y, 4UUD, and 7CHM complexes with **C10** are 3.231587e-04, 7.624966e-05, and 2.479516e-04, repectively. It means that all of our docked complexes showed low eigenvalues, indicating a considerable amount of deformability and, hence, good flexibility and stability of the molecular motion. The individual variance is shown by purple-shaded bars in the variance graph of **C10** with target proteins, while the bars with green shading show cumulative variance. The eigenvalue and variance graphs of protein-ligand complexes (H3Y3-C10, 4UUD-C10, and 7CHM-C10) are shown in Figure 14.

**FIGURE 15:**
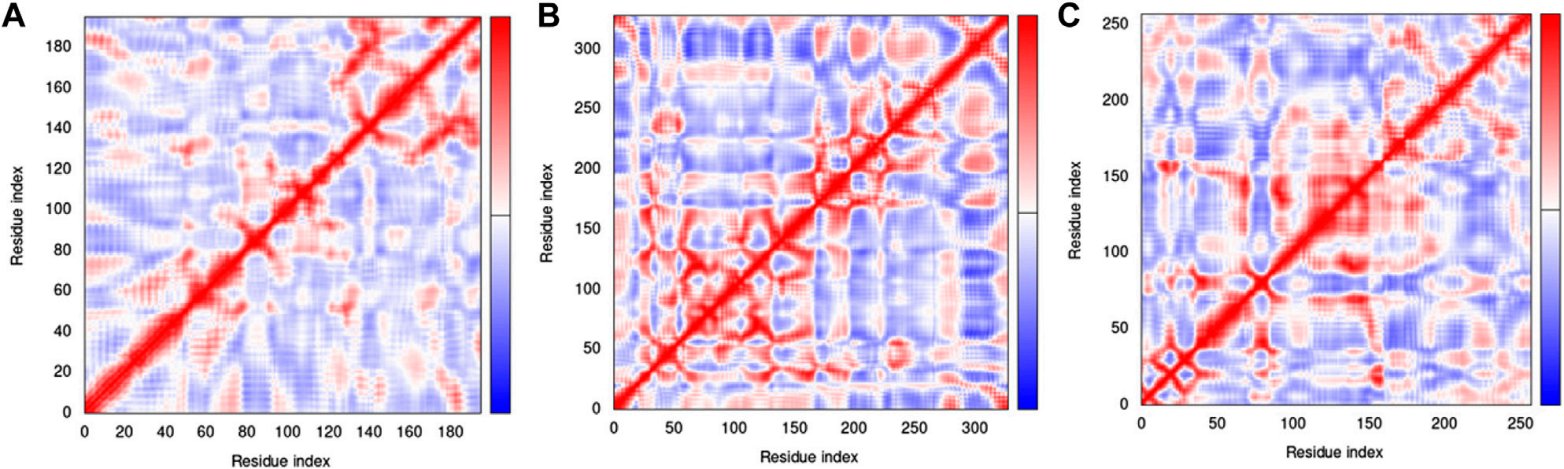
Covariance matrix of complexes **(A)** 3HY3-C10, **(B)** 4UUD-C10, and **(C)** 7CHM-C10.

The covariance matrix indicates correlations among the pairs of residues in a protein-ligand complex (Figure 15). The red and white colors showed correlated and uncorrelated motion, respectively, while anticorrelations are represented by the blue color. Greater correlation means the formation of a better complex. The covariance matrices for H3Y3-C10, 4UUD-C10, and 7CHM-C10 complexes exhibited good correlations and minimal anticorrelations.

**FIGURE 16:**
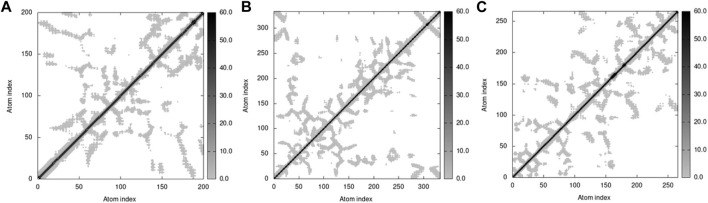
Elastic maps of docked proteins **(A)** 3HY3-C10, **(B)** 4UUD-C10, and **(C)** 7CHM-C10.

The elastic network model of docked proteins shows relationships between the atoms where the stiffer regions are indicated by the darker grey areas (Figure 16). All protein elastic maps yielded reliable results.

#### Toxicity prediction

Toxicity results provided valuable information related to the toxicological profile of selected compounds after molecular docking studies and thus may be useful for drug designing (to select the dosage and preferred route of administration (Banerjee et al., 2018). However, all these results are preliminary and must be confirmed by experiment (Table 3).

**TABLE 3:** Toxicity risk parameters.

Compounds	Hepatotoxicity	Carcinogenicity	Immunotoxicity	Mutagenicity	Cytotoxicity
**C6**	−0.60	−0.61	−0.99	+0.54	−0.86
**C10**	−0.54	−0.51	−0.99	−0.55	−0.71
**C21**	+0.56	+0.5	+0.85	+0.66	−0.56
**C76**	+0.62	+0.55	−0.99	+0.62	−0.86

As shown in Table 3, among all the hit compounds, **C10** was found to be inactive in all toxicity parameters and, thus, it was considered the best-hit compound.

### Density functional theory

#### Frontier molecular orbitals

**FIGURE 17:**
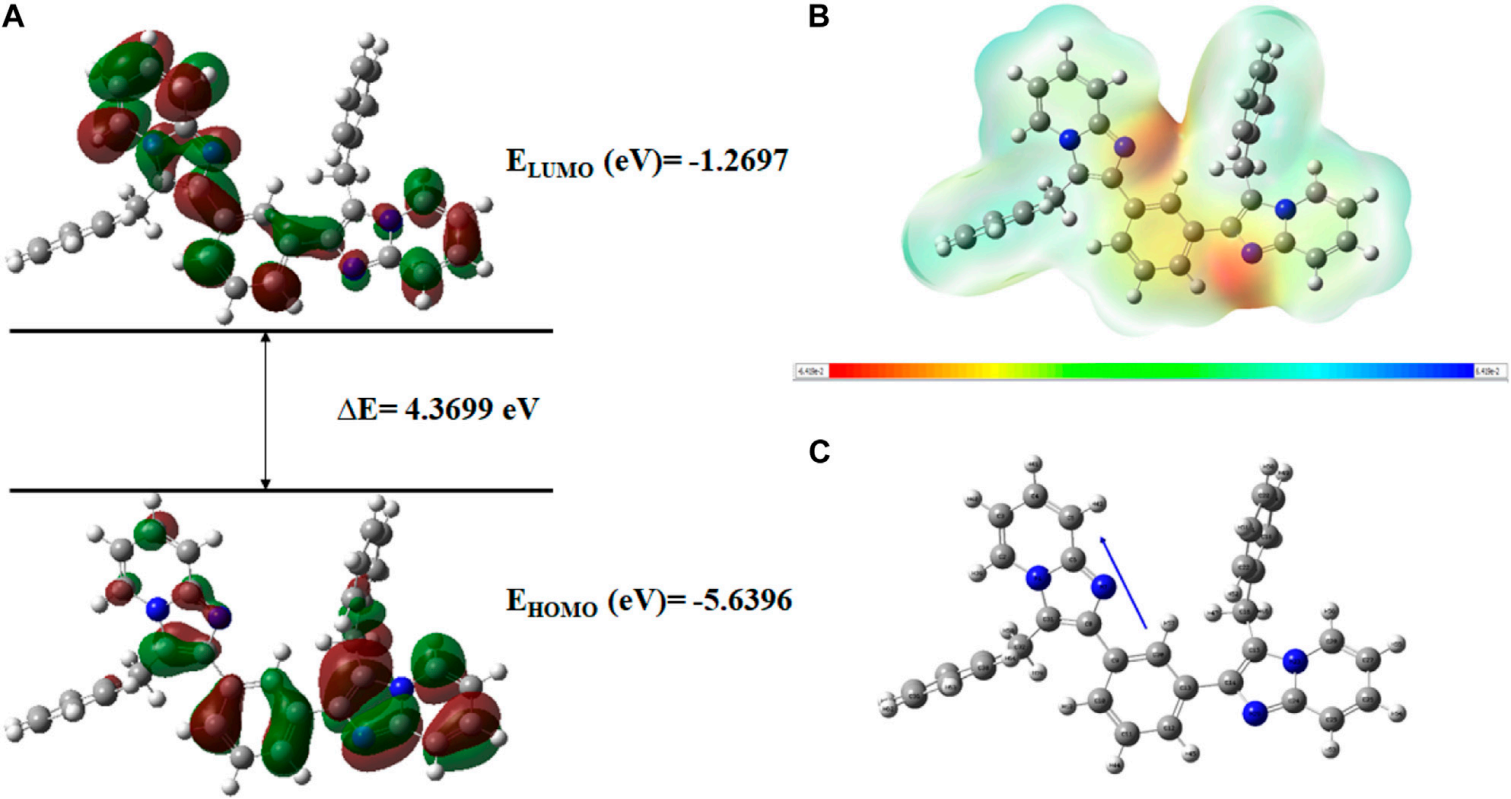
**(A)** *FMOs of* ***C6*** *along with energy gap (ΔE),* **(B)** *MEP structure and scale of* ***C6*** *based on SCF energy* ***,*** *and* **(C)** *Optimized geometry of* ***C6***.

**FIGURE 18:**
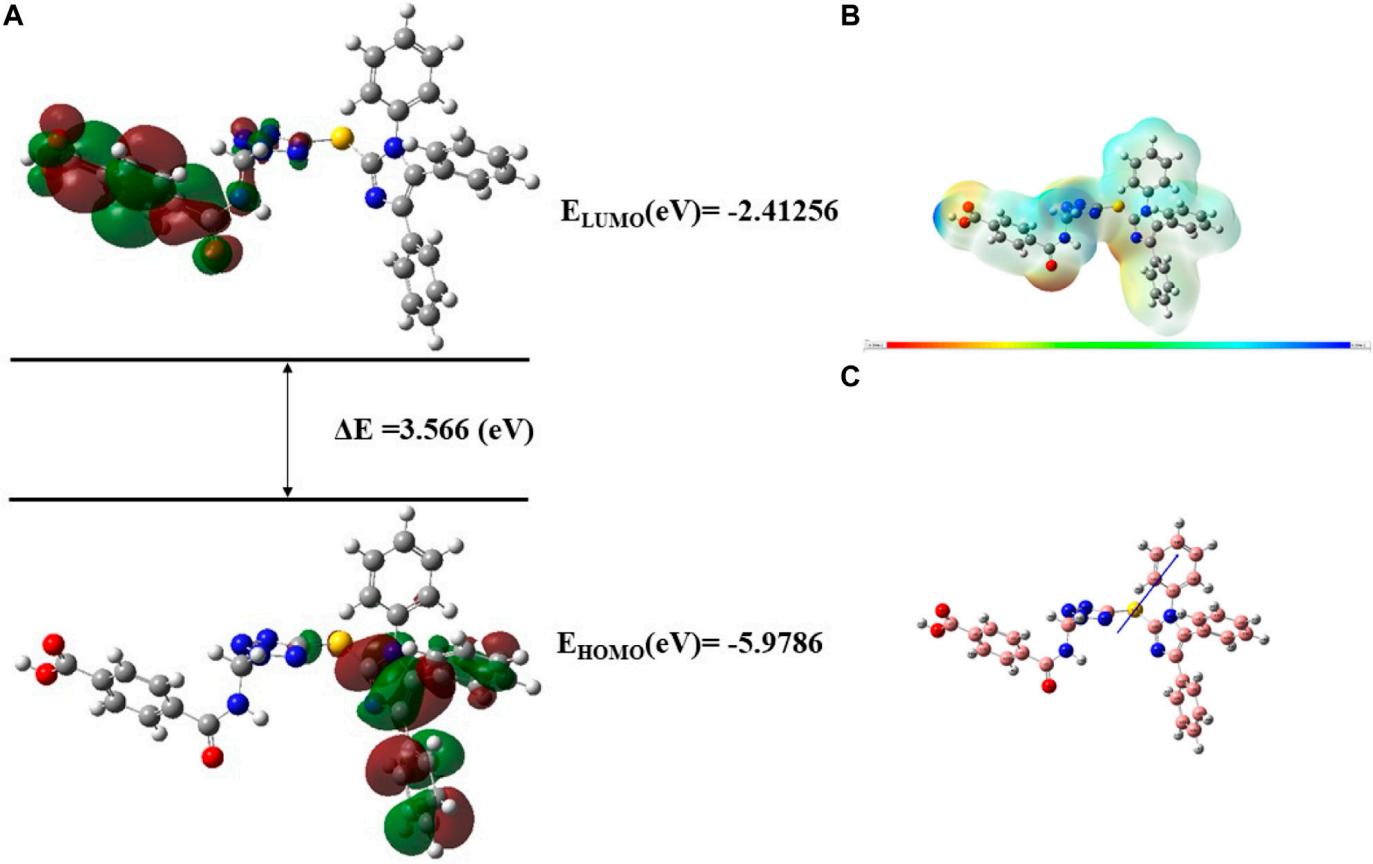
**(A)** *FMOs of* ***C10*** *along with energy gap (ΔE),* **(B)** *MEP structure and scale of* ***C10*** *based on SCF energy* ***,* (C)** *Optimized geometry of* ***C10***.

**FIGURE 19:**
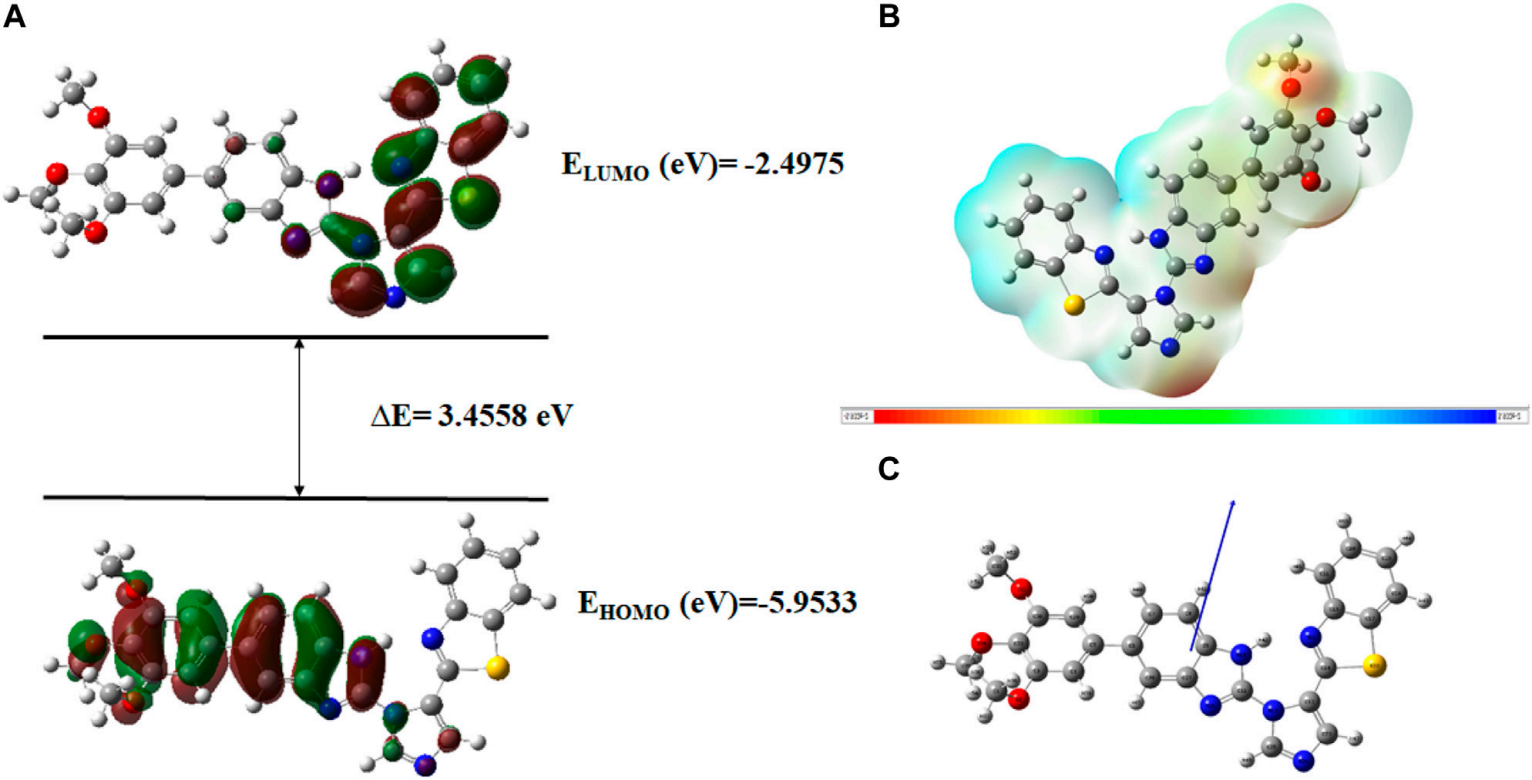
**(A)** *FMOs of* ***C21*** *along with energy gap (ΔE),* **(B)** *MEP structure and scale of* ***C21*** *based on SCF energy* ***,*** *and* **(C)** *Optimized geometry of* ***C21***.

**FIGURE 20:**
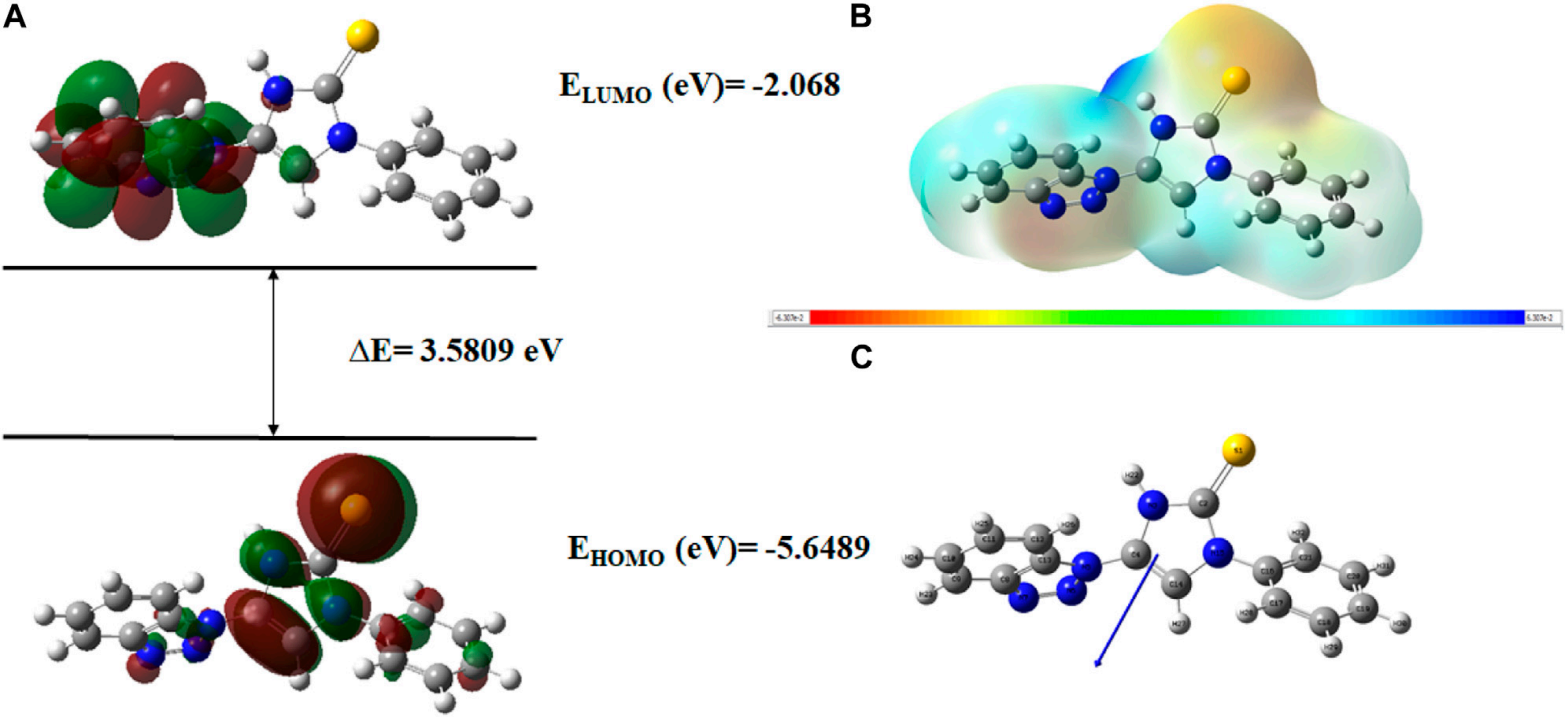
**(A)** *FMOs of* ***C76*** *along with energy gap (ΔE),* **(B)** *MEP structure and scale of* ***C76*** *based on SCF energy* ***,*** *and* **(C)** *Optimized geometry of* ***C76***.

Very useful information about the compounds can be provided by Frontier molecular orbitals (FMO) such as electronegativity, stability, reactivity, and chemical hardness and softness (Elancheran et al., 2023b). The HOMO and LUMO parameters are used to compute the chemical reactivity descriptors and to assess the molecular reactivity (Al-Janabi et al., 2021). The energy values of HOMO and LUMO were determined by the DFT method as shown in Supplementary Table S6. Contour diagrams of FMOs are shown in Figures 17–20.

The geometry optimization was done to minimize the energy and to find the most stable atomic arrangement. Optimized geometries of all compounds are shown in Figures 17–20 along with the numbering system and the vector of dipole moment.

#### Computational description

The DFT calculations for ionization energy, electron affinity, energy gap, electronegativity, chemical potential, electrophilicity index, chemical softness and hardness, additional electronic charges, maximum charger transfer index, and dipole moment were performed as explained in Supplementary Table S6).

**Table:** 

Parameters	C6	C10	C21	C76
E_HOMO_(EV)	−5.6396	−5.9786	−5.9533	−5.6489
E_lumo_(EV)	−1.2697	−2.41256	−2.4975	−2.068
ENERGY GAP ΔE (Ev)	4.3699	3.566	3.4558	3.5809
IONIZATION POTENTIAL (I = -E_HOMO_)	5.6396	5.9786	5.9533	5.6489
ELECTRON AFFINITY (A = -E_LUMO_)	1.2697	2.41256	2.4975	2.068
ELECTRONEGATIVITY (χ = $\frac{\left(I+A\right)}{2}$ ) (Ev)	3.4546	4.195	4.2254	3.8584
CHEMICAL POTENTIAL (Μ= - $\frac{\left(I+A\right)}{2}$ ) (EV)	−3.4546	−4.195	−4.2254	−3.8584
CHEMICAL HARDNESS (Η = $\frac{\left(I-A\right)}{2}$ ) (EV)	2.1849	1.78	1.7279	1.79
CHEMICAL SOFTNESS (S = $\frac{1}{2\eta }$ ) (EV)	0.2288	0.28	0.2893	0.2793
ELECTROPHILICITY INDEX (Ω = $\frac{µ2}{2\eta }$ ) (EV)	2.731	4.94	5.1663	4.1584
NUCLEOPHILICITY INDEX (N = $\frac{1}{\omega }$ ) (EV)	0.366	0.2024	0.1936	0.2405
MAXIMUM CHARGER TRANSFER INDEX (ΔN_MAX_ = $\frac{-µ}{\eta }$ ) (EV)	1.581	2.3567	2.4454	2.155
DIPOLE MOMENT (DEBYE)	3.970092	7.126807	6.225672	4.797580

A high E_HOMO_ indicates that the molecule is a strong electron donor and can easily donate electrons to the receptor, increasing biological activity. According to this concept, the activity ranking of the hit compounds is given below with an increasing E_HOMO_ value.

C10 > C21 > C76 > C6

A low E_LUMO_ value indicates that the compound can easily accept electrons from the donor molecule, increasing biological activity (Mu and Gao, 2022). According to this criterion, an increase in the biological activity of compounds is as follows:

C6 > C76 > C10 > C21

The third parameter is the energy gap (ΔE) between Homo and Lumo. If the energy gap is small, it indicates that the molecule is soft, biologically active, less stable, and has a high chemical reactivity. In other words, the biological activity increases with a decrease in the energy gap. The order of ranking should be

C21 > C10 > C76 > C6

A high chemical potential (CP) or Lower electronegativity X) value indicates electron delocalization. It means that the molecule can easily form bonds and coordinate easily with the biological system. So according to our calculated DFT data, an increase in the biological activity of compounds is given below:

C21 > C10 > C76 > C6

The dipole moment also affects the biological activity of the compound. The high value of the dipole moment indicates the strong ligand-protein interaction, thus increasing the biological activity (Sayin and Üngördü, 2018). Supplementary Table S6 shows that the dipole moment of our hit compounds are in the order of

C10 > C21 > C76 > C6

#### Molecular electrostatic potential (MEP)

In order to determine a chemical mechanism, MEP maps and MEP contours play a very important role. Molecular electrostatic potential helps to determine the hydrogen bonding interactions and to interpret the nucleophilic as well as electrophilic reactions (Horchani et al., 2020). MEP can be used to indicate the shape of the molecule and the sizes of the negative, positive, and neutral electrostatic potential. The molecular structure of drugs along with the interaction among different physicochemical properties can be predicted by the MEP scale (Al-Janabi et al., 2021). The MEP of compounds **C6, C10, C21, and C76** is determined under the basis set of B3LYP/6-311G. The negative charge is indicated by red and yellow areas that represent the electrophilic attack sites. The green color indicated a neutral charge while the blue region indicating the positively charged areas represented the nucleophilic reactivity (Bendjeddou et al., 2016). The MEP structures and MEP scales of compounds are shown in Figures 17–20.

## Conclusion

After the virtual screening, **C10** was found to be the best imidazole derivative clearing all the filters. The 3D-QSAR models generated in this study provided valuable insights into the structural features and molecular interactions that contribute to the compounds’ activity against breast cancer cells. The derived PLS regression model confirmed a fairly acceptable value of regression coefficient (*r* ^2^ = 0.81) and cross-validation regression coefficient (q^2^ = 0.51). Docking results based on the binding free energy values were found to support the best-hit compounds. The DFT calculations also confirm the best alternative cancer inhibitor. These predictions aided in rationalizing the observed biological activities and potential mechanisms of action of these compounds against breast cancer cells. MD simulation study supported the docking results of **C10** to its target proteins or receptors. This study showcased the lead compound’s stability and robustness, suggesting its suitability for further preclinical and clinical evaluations. The compound’s favorable binding profile, coupled with its ability to sustain its interactions over extended simulation periods, instills confidence in its potential as a promising candidate for subsequent stages of drug development. The results acquired from the present study may be utilized in the future to develop more imidazole-based therapeutics against cancer. The identification of Compound C10 as a lead compound opens up avenues for further drug development and optimization and offers valuable insights and potential directions for future research and clinical applications.

## Data Availability

The datasets presented in this study can be found in online repositories. The names of the repository/repositories and accession number(s) can be found in the article/Supplementary Material.
