# Supplementary material for: The combination of multi-approach studies to explore the potential therapeutic mechanisms of imidazole derivatives as an MCF-7 inhibitor in therapeutic strategies
Source: Front Chem. 2023 Jun 27;11:1197665. doi: 10.3389/fchem.2023.1197665 (PMC10335751; doi:10.3389/fchem.2023.1197665)
Supplement: Supplementary file 1 [file DataSheet1.docx]

**Combination of multi-approach Study to explore the Potential Therapeutic Mechanisms of imidazole derivatives as MCF-7 inhibitor in therapeutic strategies**

**Maryam Rashid^1^, Ayesha Maqbool^1^, Nusrat Shafiq^1^*, Yousef A. Bin Jardan^2^, Shagufta Parveen^1,3^, Mohammed Bourhia^4^, Hiba-Allah Nafidi^5^, Rashid Ahmed Khan^6^**

^1^Synthetic and Natural Product Drug Discovery Laboratory, Department of Chemistry, Government College Women University Faisalababd-38000, Pakistan

^2^Department of Pharmaceutics, College of Pharmacy, King Saud University, Riyadh, Saudi Arabia

^3^Department of Applied Chemistry, Beijing Institute of Technology, No. 5, Zhongguancun South Street, 100081 Beijing, China

^4^ Department of Chemistry and Biochemistry, Faculty of Medicine and Pharmacy, Ibn Zohr University, Laayoune 70000, Morocco

^5^Department of Food Science, Faculty of Agricultural and Food Sciences, Laval University, 2325 Quebec City, QC G1V 0A6, Canada

^6^Nuclear Institute for Agriculture & Biology (NIAB), Faisalabad-38000, Pakistan

**Corresponding Author: Dr. Nusrat Shafiq**

**Corresponding Address:** [dr.nusratshafiq@gcwuf.edu.pk](mailto:dr.nusratshafiq@gcwuf.edu.pk); [gqumarin@gmail.com](mailto:gqumarin@gmail.com);

**ORCID: orcid.org/0000-0002-3270-4227**

***Supplementary material***


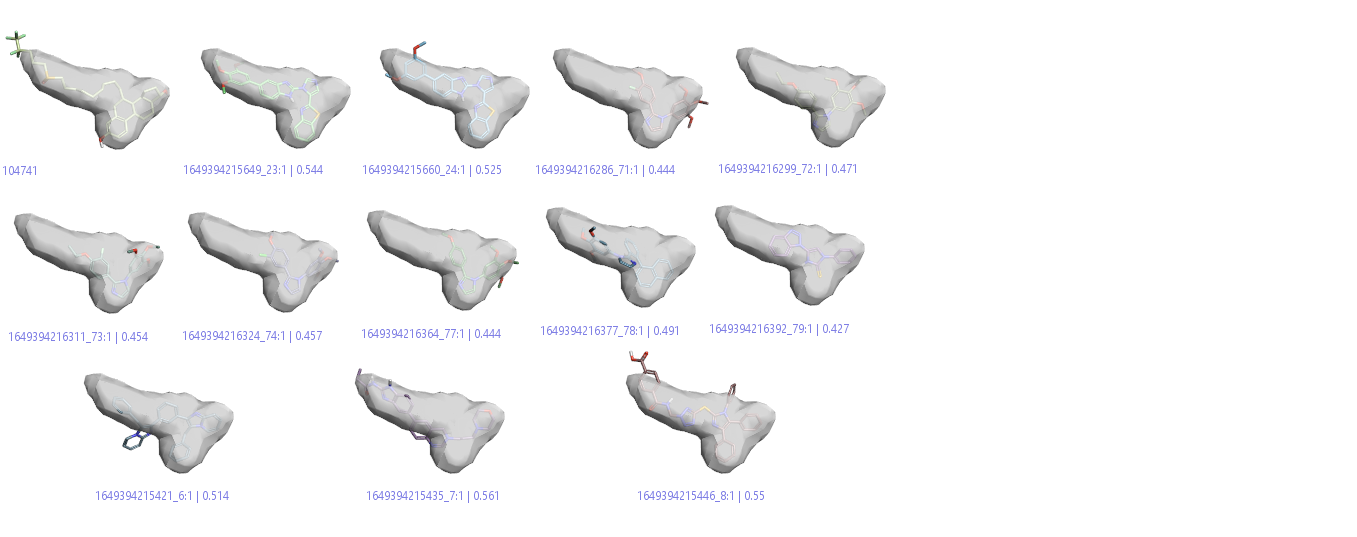


*Figure S1: Shape regions of active compounds*


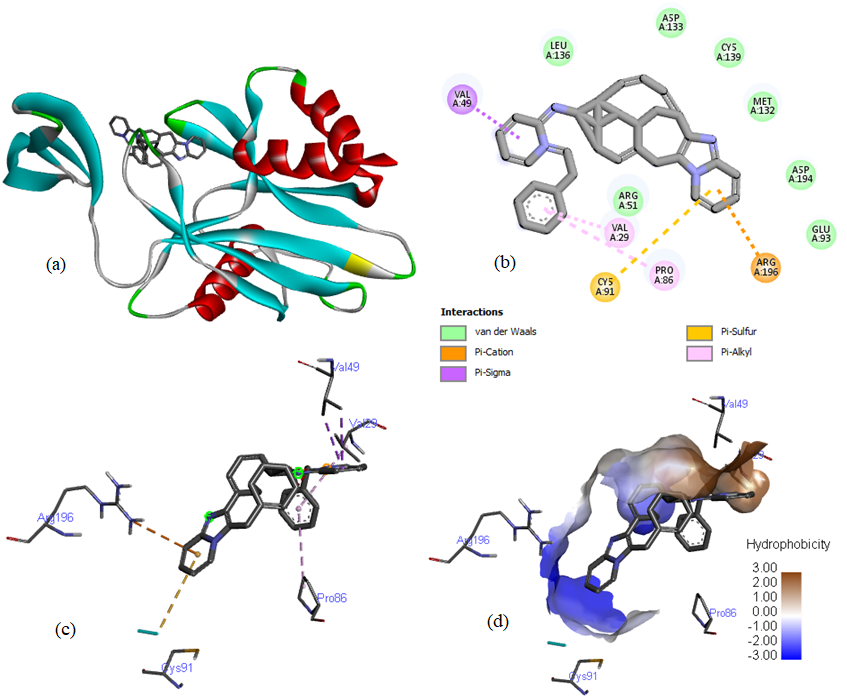


*Fig. S2: Molecular docking of compound* ***C6*** *with PDB ID: 5NWH, (a) 3D view of the best selected conformation, (b) 2D Interactions, (c) Ligand interactions, (d) Hydrophobicity*


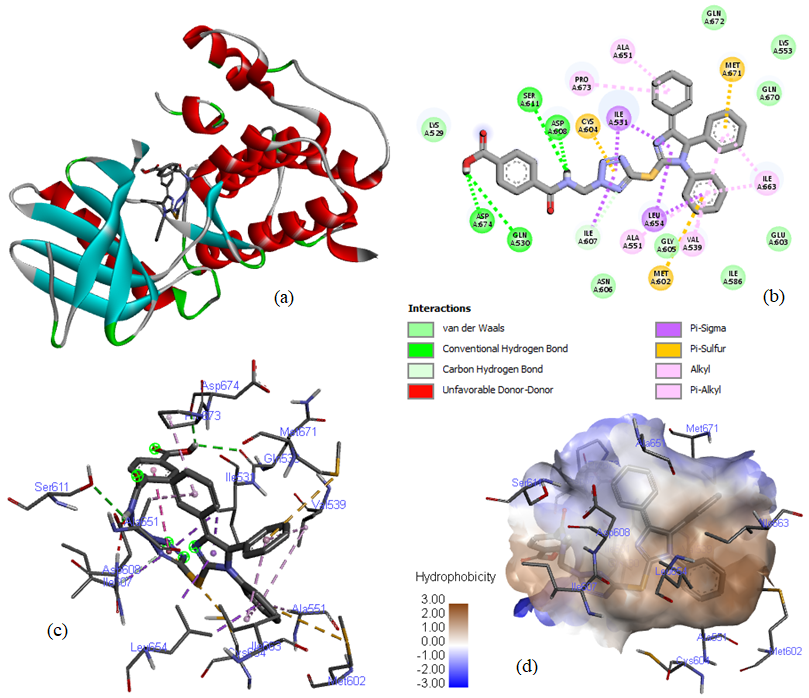


*Figure S3: Molecular docking of compound* ***C10*** *with PDB ID: 7CHM, (a) 3D view of the best selected conformation, (b) 2D Interactions, (c) Ligand interactions, (d) Hydrophobicity*


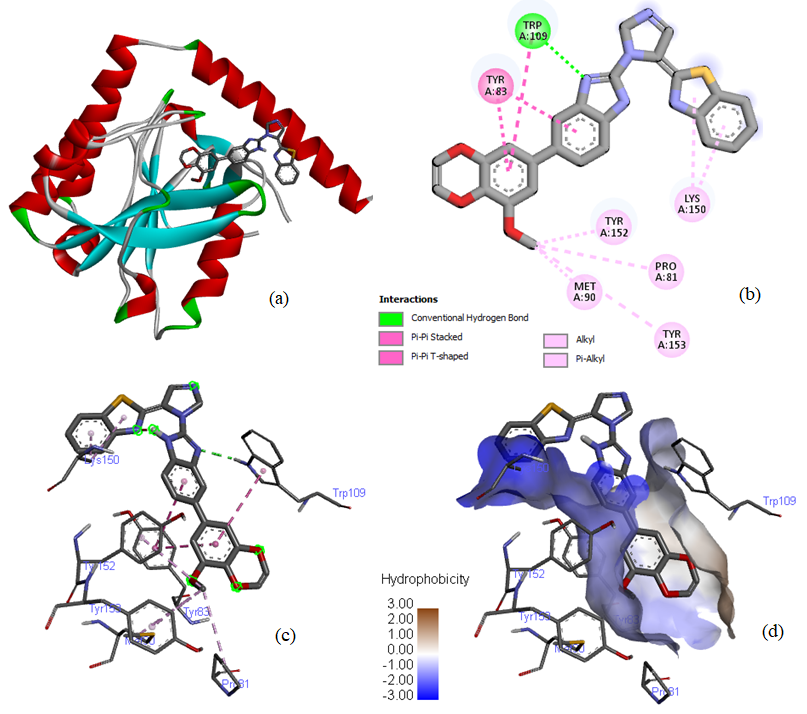


*Fig. S4: Molecular docking of compound* ***C21*** *with PDB ID: 3HY3 (a) 3D view of the best selected conformation, (b) 2D Interactions, (c) Ligand interactions, (d) Hydrophobicity*

**
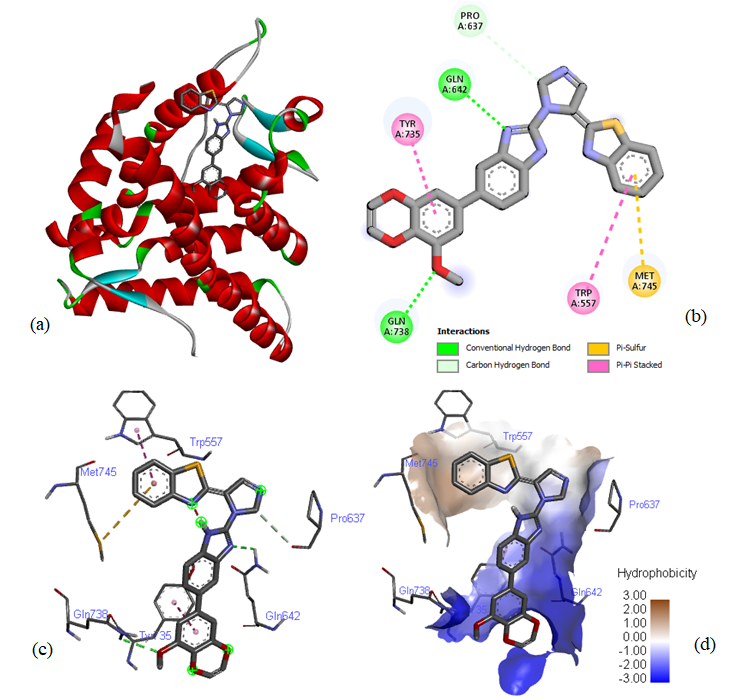
**

*Figure S5: Molecular docking of compound C21 with PDB ID: 4UDD (a) 3D view of the best selected conformation. (b) 2D Interactions. (c) Ligand interactions. (d) Hydrophobicity*

***Table S-1: Data set library of imidazole derivatives***

| **Accession No.** | **Structure** | **Smiles** | **IUPAC Names** | **IC50 value (µM)** | **References** |
| --- | --- | --- | --- | --- | --- |
| **C1** |  | ClC(C=C1)=CC=C1C(N2)=C(NC(C)(C)C)N3C2=C(C(OCC)=O)C=N3 | Ethyl 3-(tert-butylamino)-2-(4-chlorophenyl)-5H-imidazo[1,2-b]pyrazole-7-carboxylate | 15 | (Baviskar *et al.*, 2011). |
| **C2** |  | CN1C(C([O-])=O)=C(NC1)C([O-])=O.[NH3+]CC2(C(CCC3=C4C=CC(C(C)C)=C3)C4(C)CCC2)C.[NH3+]CC5(CCCC6(C)C5CCC7=C6C=CC(C(C)C)=C7)C | N-1-methyl-1H-imidazole-4,5-diamino-carboxy salt-dehydroabietylamine | 0.75±0.07 | (Zhao *et al.*, 2018). |
| **C3** |  | NCC1=CC=C(NC2=NC3=NC=CN3C(NC4=CC=CC=C4C(NC)=O)=N2)C=C1 | 2-[[2-[4-(aminomethyl)anilino]imidazo[1,2-a][1,3,5]triazin-4-yl]amino]-N-methylbenzamide | 1.08±0.45 µM | (Dao *et al.*, 2015). |
| **C4** |  | S=C(NC(CC1=CC=CC=C1)=O)N2C(C3=CC=CC=C3)=NC4=CC=CC=C42 | 2-phenyl-N-(2-phenyl-1H-benzo[d]imidazole-1-carbonothioyl)acetamide | 8.32±0.04 | (Hashem *et al.*, 2022). |
| **C5** |  | O=C(NC1=NC2=CC=CC=C2N1)CC3=CC=CC=C3 | N-(1H-Benzo[d]imidazol-2-yl)-2-phenylacetamide | 6.59±0.07 | (Hashem *et al.*, 2022). |
| **C6** |  | N12C=CC=CC1=NC(C3=CC=CC(C4=C(CC5=CC=CC=C5)N6C(C=CC=C6)=N4)=C3)=C2CC7=CC=CC=C7 | 1,3-bis(3-benzylimidazo[1,2-a]pyridin-2-yl)benzene | 0.30 | (Meenakshisundaram *et al.*, 2019). |
| **C7** |  | CCC(NC1=NC2=C(C=CC(C3=CC=C4C(CN(CCCN5CCOCC5)C=N4)=C3)=C2)N1)=O | N-(5-(3-(3-morpholinopropyl)-3,4-dihydroquinazolin-6-yl)-1H-benzo[d]imidazol-2-yl)propionamide | 0.38±0.08 | (Fan *et al.*, 2020). |
| **C8** |  | S=C1NC=C(C2=CC=C(OC)C=C2)N1/N=C/C3=CC=CC=C3O | (E)-1-(2-Hydroxybenzylideneamino)-5-(4-methoxyphenyl)-1H-imidazole-2(3H)- thione | 1.071±0.1 | (Abu Almaaty *et al.*, 2021). |
| **C9** |  | S=C1NC=C(C2=CC=C(Cl)C=C2)N1/N=C/C3=CC=C(OC)C=C3 | (E)-5-(4-Chlorophenyl)-1-(4-methoxybenzylideneamino)-1H-imidazole-2(3H)-thione | 7.967 ± 0.5 | (Abu Almaaty *et al.*, 2021). |
| **C10** |  | O=C(C1=CC=C(C(O)=O)C=C1)NCN(N=N2)N=C2SC3=NC(C4=CC=CC=C4)=C(C5=CC=CC=C5)N3C6=CC=CC=C6 | 4-(((5-((1,4,5-triphenyl-1H-imidazol-2-yl)thio)-2H-tetrazol-2-yl)methyl)carbamoyl)benzoic acid | 0.38±0.04 | (Al-Blewi *et al.*, 2021). |
| **C11** |  | ClC1=C(Cl)C=C(N=C(S(C)=O)N2C#CC#CC)C2=C1.[HH].[HH].[HH] | 5,6-dichloro-2-(methylsulfinyl)-1-(penta-1,3-diyn-1-yl)-1H-benzo[d]imidazole--dihydrogen (1/3) | 0.5 | (Ali *et al.*, 2017). |
| **C12** |  | CC1=C(C)C=C(N=C(S(=O)(C)=O)N2C#CC#CC)C2=C1.[HH].[HH].[HH] | 5,6-dimethyl-2-(methylsulfonyl)-1-(penta-1,3-diyn-1-yl)-1H-benzo[d]imidazole--dihydrogen (1/3) | 0.6 | (Ali *et al.*, 2017). |
| **C13** |  | CC1=NC2=CC([N+]([O-])=O)=CC=C2N1 | 2-methyl-6-nitro-1H-benzimidazole | 4.52 | (Ali *et al.*, 2017). |
| **C14** |  | CC1=NC2=CC([N+]([O-])=O)=CC=C2N1CC3=NN=C(SCC(C)=O)S3 | 1-((5-((2-methyl-5-nitro-1H-benzo[d]imidazol-1-yl)methyl)-1,3,4-thiadiazol-2-yl)thio)propan-2-one | 8.29 | (Ali *et al.*, 2017). |
| **C15** |  | CN1C(C2=CC=CC=C2)=C(C3=CC=CC=C3)N=C1C4=CC=C(NC(CSC5=NC6=C(C=CC=C6)O5)=O)C=C4 | 2-(1,3-benzoxazol-2-ylsulfanyl)-N-[4-(1-methyl-4,5-diphenylimidazol-2-yl)phenyl]acetamide | 11.2 | (Özkay *et al.*, 2010). |
| **C16** |  | CN1C(C2=CC=CC=C2)=C(C3=CC=CC=C3)N=C1C4=CC=C(NC(CSC5=NN=CN5C)=O)C=C4 | N-[4-(1-methyl-4,5-diphenylimidazol-2-yl)phenyl]-2-[(4-methyl-1,2,4-triazol-3-yl)sulfanyl]acetamide | 3.2 | (Özkay *et al.*, 2010). |
| **C17** |  | CN1C(C2=CC=CC=C2)=C(C3=CC=CC=C3)N=C1C4=CC=C(NC(CSC5=NN=NN5C)=O)C=C4 | N-[4-(1-methyl-4,5-diphenylimidazol-2-yl)phenyl]-2-(1-methyltetrazol-5-yl)sulfanylacetamide | 4.5 | (Özkay *et al.*, 2010). |
| **C18** |  | CN1C(C2=CC=CC=C2)=C(C3=CC=CC=C3)N=C1C4=CC=C(NC(CSC5=NN=C(C)S5)=O)C=C4 | N-[4-(1-methyl-4,5-diphenylimidazol-2-yl)phenyl]-2-[(5-methyl-1,3,4-thiadiazol-2-yl)sulfanyl]acetamide | 3.2 | (Özkay *et al.*, 2010). |
| **C19** |  | CN1C(C2=CC=CC=C2)=C(C3=CC=CC=C3)N=C1C4=CC=C(NC(CN5CCN(C6=CC=CC=C6)CC5)=O)C=C4 | N-[4-(1-methyl-4,5-diphenylimidazol-2-yl)phenyl]-2-(4-phenylpiperazin-1-yl)acetamide | 10.7 | (Özkay *et al.*, 2010). |
| **C20** |  | C1(C2=CC=C(NC(N3C(C4=NC5=C(C=CC=C5)S4)=CN=C3)=N6)C6=C2)=CC=CC=C1 | 2-(1-(5-phenyl-1H-benzo[d]imidazol-2-yl)-1H-imidazol-5-yl)benzo[d]thiazole | 2.89±1.77 | (Edukondalu *et al.*, 2021). |
| **C21** |  | COC1=CC(C2=CC=C(NC(N3C(C4=NC5=C(C=CC=C5)S4)=CN=C3)=N6)C6=C2)=CC(OC)=C1OC | 2-(1-(5-(3,4,5-trimethoxyphenyl)-1H-benzo[d]imidazol-2-yl)-1H-imidazol-5-yl)benzo[d]thiazole | 0.018±0.0039 | (Edukondalu *et al.*, 2021). |
| **C22** |  | COC1=CC(C2=CC=C(NC(N3C(C4=NC5=C(C=CC=C5)S4)=CN=C3)=N6)C6=C2)=CC(OC)=C1 | 2-(1-(5-(3,5-dimethoxyphenyl)-1H-benzo[d]imidazol-2-yl)-1H-imidazol-5-yl)benzo[d]thiazole | 0.10±0.028 | (Edukondalu *et al.*, 2021). |
| **C23** |  | COC1=CC=C(C2=CC=C(NC(N3C(C4=NC5=C(C=CC=C5)S4)=CN=C3)=N6)C6=C2)C=C1 | 2-(1-(5-(4-methoxyphenyl)-1H-benzo[d]imidazol-2-yl)-1H-imidazol-5-yl)benzo[d]thiazole | 1.22±0.36 | (Edukondalu *et al.*, 2021). |
| **C24** |  | ClC1=CC=C(C2=CC=C(NC(N3C(C4=NC5=C(C=CC=C5)S4)=CN=C3)=N6)C6=C2)C=C1 | 2-(1-(5-(4-chlorophenyl)-1H-benzo[d]imidazol-2-yl)-1H-imidazol-5-yl)benzo[d]thiazole | 4.77±2.44 | (Edukondalu *et al.*, 2021). |
| **C25** |  | BrC1=CC=C(C2=CC=C(NC(N3C(C4=NC5=C(C=CC=C5)S4)=CN=C3)=N6)C6=C2)C=C1 | 2-(1-(5-(4-bromophenyl)-1H-benzo[d]imidazol-2-yl)-1H-imidazol-5-yl)benzo[d]thiazole | 3.94±1.98 | (Edukondalu *et al.*, 2021). |
| **C26** |  | O=[N+](C1=CC=C(C2=CC=C(NC(N3C(C4=NC5=C(C=CC=C5)S4)=CN=C3)=N6)C6=C2)C=C1)[O-] | 2-(1-(5-(4-nitrophenyl)-1H-benzo[d]imidazol-2-yl)-1H-imidazol-5-yl)benzo[d]thiazole | 1.10±0.23 | (Edukondalu *et al.*, 2021). |
| **C27** |  | CC1=CC=C(C2=CC=C(NC(N3C(C4=NC5=C(C=CC=C5)S4)=CN=C3)=N6)C6=C2)C=C1 | 2-(1-(5-(p-tolyl)-1H-benzo[d]imidazol-2-yl)-1H-imidazol-5-yl)benzo[d]thiazole | 2.00±1.35 | (Edukondalu *et al.*, 2021). |
| **C28** |  | CN(C)NC1=CC=C(C2=CC=C(NC(N3C(C4=NC5=C(C=CC=C5)S4)=CN=C3)=N6)C6=C2)C=C1 | 2-(1-(5-(4-(2,2-dimethylhydrazineyl)phenyl)-1H-benzo[d]imidazol-2-yl)-1H-imidazol-5-yl)benzo[d]thiazole | 2.99±1.78 | (Edukondalu *et al.*, 2021). |
| **C29** |  | CCCCN(N=N1)C=C1CSC2=NC(C3=CC=CC=C3)=C(C4=CC=CC=C4)N2C5=CC=CC=C5 | 1-tridecyl-4-(((1,4,5-triphenyl-1H-imidazol-2-yl)thio)methyl)-1H-1,2,3-triazole | 0.98±0.05 | (Al-Blewi *et al.*, 2021). |
| **C30** |  | CCCCN(N=N1)C=C1CSC2=NC(C3=CC=CC=C3)=C(C4=CC=CC=C4)N2C5=CC=CC=C5 | 1-icosyl-4-(((1,4,5-triphenyl-1H-imidazol-2-yl)thio)methyl)-1H-1,2,3-triazole | 3.21±0.02 | (Al-Blewi *et al.*, 2021). |
| **C31** |  | O=C(C1=CC=C(Cl)C(Cl)=C1)NCCN(N=N2)C=C2CSC3=NC(C4=CC=CC=C4)=C(C5=CC=CC=C5)N3C6=CC=CC=C6 | 3,4-dichloro-N-(2-(4-(((1,4,5-triphenyl-1H-imidazol-2-yl)thio)methyl)-1H-1,2,3-triazol-1-yl)ethyl)benzamide | 6.41±0.18 | (Al-Blewi *et al.*, 2021). |
| **C32** |  | O=C(C1=CC=C([N+]([O-])=O)C=C1)NCCN(N=N2)C=C2CSC3=NC(C4=CC=CC=C4)=C(C5=CC=CC=C5)N3C6=CC=CC=C6 | 4-nitro-N-(2-(4-(((1,4,5-triphenyl-1H-imidazol-2-yl)thio)methyl)-1H-1,2,3-triazol-1-yl)ethyl)benzamide | 9.46±0.27 | (Al-Blewi *et al.*, 2021). |
| **C33** | **** | O=C(C1=CC=CC=C1F)NCCN(N=N2)C=C2CSC3=NC(C4=CC=CC=C4)=C(C5=CC=CC=C5)N3C6=CC=CC=C6 | 2-fluoro-N-(2-(4-(((1,4,5-triphenyl-1H-imidazol-2-yl)thio)methyl)-1H-1,2,3-triazol-1-yl)ethyl)benzamide | 3.80±0.12 | (Al-Blewi *et al.*, 2021). |
| **C34** | **** | O=C(C1=CC=C(F)C=C1)NCCN(N=N2)C=C2CSC3=NC(C4=CC=CC=C4)=C(C5=CC=CC=C5)N3C6=CC=CC=C6 | 4-fluoro-N-(2-(4-(((1,4,5-triphenyl-1H-imidazol-2-yl)thio)methyl)-1H-1,2,3-triazol-1-yl)ethyl)benzamide | 4.45±0.08 | (Al-Blewi *et al.*, 2021). |
| **C35** | **** | O=C(C1=CC=C(C(O)=O)C=C1)NCCN(N=N2)C=C2CSC3=NC(C4=CC=CC=C4)=C(C5=CC=CC=C5)N3C6=CC=CC=C6 | 4-((2-(4-(((1,4,5-triphenyl-1H-imidazol-2-yl)thio)methyl)-1H-1,2,3-triazol-1-yl)ethyl)carbamoyl)benzoic acid | 0.38±0.04 | (Al-Blewi *et al.*, 2021). |
| **C36** | **** | O=C(C(C=C1)=CC=C1N(N=N2)C=C2CSC3=NC(C4=CC=CC=C4)=C(C5=CC=CC=C5)N3C6=CC=CC=C6)O | 4-(4-(((1,4,5-triphenyl-1H-imidazol-2-yl)thio)methyl)-1H-1,2,3-triazol-1-yl)benzoic acid | 3.87±0.07 | (Al-Blewi *et al.*, 2021). |
| **C37** | **** | O=C1N(CC)C(CCN2CCCCC2)=NC(C1=C3)=CC=C3C4=CC5=C(N=C(NC(CC)=O)N5)C=C4 | N-(6-(3-ethyl-4-oxo-2-(2-(piperidin-1-yl)ethyl)-3,4-dihydroquinazolin-6-yl)-1H-benzo[d]imidazol-2-yl)propionamide | 0.94±0.14 | (Fan *et al.*, 2020). |
| **C38** | **** | O=C1N(CCCN2CCCCC2)C=NC(C1=C3)=CC=C3C4=CC5=C(N=C(NC(CC)=O)N5)C=C4 | N-(6-(4-oxo-3-(3-(piperidin-1-yl)propyl)-3,4-dihydroquinazolin-6-yl)-1H-benzo[d]imidazol-2-yl)propionamide | 1.23±0.09 | (Fan *et al.*, 2020). |
| **C39** | **** | O=C1N(CCCN2CCCCC2)C=NC(C1=C3)=CC=C3C4=CC5=C(N=C(NC(CCCC)=O)N5)C=C4 | N-(6-(4-oxo-3-(3-(piperidin-1-yl)propyl)-3,4-dihydroquinazolin-6-yl)-1H-benzo[d]imidazol-2-yl)pentanamide | 3.85±0.28 | (Fan *et al.*, 2020). |
| **C40** | **** | O=C1N(CCCCN2CCCCC2)C=NC(C1=C3)=CC=C3C4=CC5=C(N=C(NC(CC)=O)N5)C=C4 | N-(6-(4-oxo-3-(4-(piperidin-1-yl)butyl)-3,4-dihydroquinazolin-6-yl)-1H-benzo[d]imidazol-2-yl)propionamide | 1.22±0.23 | (Fan *et al.*, 2020). |
| **C41** | **** | O=C1N(CCCN2CCCCC2)C=NC(C1=C3)=CC=C3C4=CC5=C(N=C(NC(N(C)C)=O)N5)C=C4 | 1,1-dimethyl-3-(6-(4-oxo-3-(3-(piperidin-1-yl)propyl)-3,4-dihydroquinazolin-6-yl)-1H-benzo[d]imidazol-2-yl)urea | 4.85±0.69 | (Fan *et al.*, 2020). |
| **C42** | **** | O=C1N(CCCN2CCCCC2)C=NC(C1=C3)=CC=C3C4=CC5=C(N=C(NC(N(CC)CC)=O)N5)C=C4 | 1,1-diethyl-3-(6-(4-oxo-3-(3-(piperidin-1-yl)propyl)-3,4-dihydroquinazolin-6-yl)-1H-benzo[d]imidazol-2-yl)urea | 1.76±0.17 | (Fan *et al.*, 2020). |
| **C43** | **** | O=C1N(CCCN2CCCCC2)C=NC(C1=C3)=CC=C3C4=CC5=C(N=C(NC(C6CC6)=O)N5)C=C4 | N-(6-(4-oxo-3-(3-(piperidin-1-yl)propyl)-3,4-dihydroquinazolin-6-yl)-1H-benzo[d]imidazol-2-yl)cyclopropanecarboxamide | 1.61±0.21 | (Fan *et al.*, 2020). |
| **C44** | **** | O=C1N(CCCC2=CNC3=C2C=CC=C3)C=NC(C1=C4)=CC=C4C5=CC6=C(N=C(NC(CC)=O)N6)C=C5 | N-(6-(3-(3-(1H-indol-3-yl)propyl)-4-oxo-3,4-dihydroquinazolin-6-yl)-1H-benzo[d]imidazol-2-yl)propionamide | 0.55±0.10 | (Fan *et al.*, 2020). |
| **C45** | **** | O=C(C1=CC=C(Br)C=C1)C[N+](C=C2)=CN2C(C3=CC=CC=C3)C4=CC5=CC=CC=C5O4.[Br-] | 2-[3-[1-benzofuran-2-yl(phenyl)methyl]imidazol-1-ium-1-yl]-1-(4-bromophenyl)ethanone;bromide | 11.99 | (Wang *et al.*, 2013). |
| **C46** | **** | O=C(C1=CC=C(C=CC=C2)C2=C1)C[N+](C=C3)=CN3C(C4=CC=CC=C4)C5=CC6=CC=CC=C6O5.[Br-] | 2-[3-[1-benzofuran-2-yl(phenyl)methyl]imidazol-1-ium-1-yl]-1-naphthalen-2-ylethanone;bromide | 3.12 | (Wang *et al.*, 2013). |
| **C47** | **** | O=C(C1=CC=CC=C1)C[N+](C=C2)=C(C)N2C(C3=CC=CC=C3)C4=CC5=CC=CC=C5O4.[Br-] | 2-[3-[1-benzofuran-2-yl(phenyl)methyl]-2-methylimidazol-1-ium-1-yl]-1-phenylethanone;bromide | 10.24 | (Wang *et al.*, 2013). |
| **C48** | **** | O=C(C1=CC=C(F)C=C1)C[N+](C=C2)=C(C)N2C(C3=CC=CC=C3)C4=CC5=CC=CC=C5O4.[Br-] | 2-[3-[1-benzofuran-2-yl(phenyl)methyl]-2-methylimidazol-1-ium-1-yl]-1-(4-fluorophenyl)ethanone;bromide | 10.70 | (Wang *et al.*, 2013). |
| **C49** | **** | O=C(C1=CC=C(OC)C=C1)C[N+](C=C2)=C(C)N2C(C3=CC=CC=C3)C4=CC5=CC=CC=C5O4.[Br-] | 2-[3-[1-benzofuran-2-yl(phenyl)methyl]-2-methylimidazol-1-ium-1-yl]-1-(4-methoxyphenyl)ethanone;bromide | 9.84 | (Wang *et al.*, 2013). |
| **C50** | **** | O=C(C1=CC=C(C=CC=C2)C2=C1)C[N+](C=C3)=C(C)N3C(C4=CC=CC=C4)C5=CC6=CC=CC=C6O5.[Br-] | 2-[3-[1-benzofuran-2-yl(phenyl)methyl]-2-methylimidazol-1-ium-1-yl]-1-naphthalen-2-ylethanone;bromide | 2.24 | (Wang *et al.*, 2013). |
| **C51** | **** | CCC1=[N+](CCC2=CC=CC=C2)C=CN1C(C3=CC=CC=C3)C4=CC5=CC=CC=C5O4.[Br-] | 1-(benzofuran-2-yl(phenyl)methyl)-2-ethyl-3-phenethyl-1H-imidazol-3-ium;bromide | 5.24 | (Wang *et al.*, 2013). |
| **C52** | **** | O=C(C1=CC=CC=C1)C[N+](C=C2)=C(CC)N2C(C3=CC=CC=C3)C4=CC5=CC=CC=C5O4.[Br-] | 2-[3-[1-benzofuran-2-yl(phenyl)methyl]-2-ethylimidazol-1-ium-1-yl]-1-phenylethanone;bromide | 13.96 | (Wang *et al.*, 2013). |
| **C53** | **** | O=C(C1=CC=C(F)C=C1)C[N+](C=C2)=C(CC)N2C(C3=CC=CC=C3)C4=CC5=CC=CC=C5O4.[Br-] | 2-[3-[1-benzofuran-2-yl(phenyl)methyl]-2-ethylimidazol-1-ium-1-yl]-1-(4-fluorophenyl)ethanone;bromide | 13.77 | (Wang *et al.*, 2013). |
| **C54** | **** | O=C(C1=CC=C(Br)C=C1)C[N+](C=C2)=C(CC)N2C(C3=CC=CC=C3)C4=CC5=CC=CC=C5O4.[Br-] | 2-[3-[1-benzofuran-2-yl(phenyl)methyl]-2-ethylimidazol-1-ium-1-yl]-1-(4-bromophenyl)ethanone;bromide | 9.50 | (Wang *et al.*, 2013). |
| **C55** | **** | O=C(C1=CC=C(OC)C=C1)C[N+](C=C2)=C(CC)N2C(C3=CC=CC=C3)C4=CC5=CC=CC=C5O4.[Br-] | 2-[3-[1-benzofuran-2-yl(phenyl)methyl]-2-ethylimidazol-1-ium-1-yl]-1-(4-methoxyphenyl)ethanone;bromide | 9.50 | (Wang *et al.*, 2013). |
| **C56** | **** | O=C(C1=CC=C(C=CC=C2)C2=C1)C[N+](C=C3)=C(CC)N3C(C4=CC=CC=C4)C5=CC6=CC=CC=C6O5.[Br-] | 2-[3-[1-benzofuran-2-yl(phenyl)methyl]-2-ethylimidazol-1-ium-1-yl]-1-naphthalen-2-ylethanone;bromide | 4.20 | (Wang *et al.*, 2013). |
| **C57** | **** | C12=CC=CC=C1C=C(C(C3=CC=CC=C3)N4C=[N+](CCC5=CC=CC=C5)C6=C4C=CC=C6)O2.[Br-] | 1-(benzofuran-2-yl(phenyl)methyl)-3-phenethyl-1H-benzo[d]imidazol-3-ium;bromide | 9.16 | (Wang *et al.*, 2013). |
| **C58** | **** | O=C(C1=CC=CC=C1)C[N+](C2=C3C=CC=C2)=CN3C(C4=CC=CC=C4)C5=CC6=CC=CC=C6O5.[Br-] | 2-[3-[1-benzofuran-2-yl(phenyl)methyl]benzimidazol-1-ium-1-yl]-1-phenylethanone;bromide | 9.59 | (Wang *et al.*, 2013). |
| **C59** | **** | O=C(C1=CC=C(F)C=C1)C[N+](C2=C3C=CC=C2)=CN3C(C4=CC=CC=C4)C5=CC6=CC=CC=C6O5.[Br-] | 2-[3-[1-benzofuran-2-yl(phenyl)methyl]benzimidazol-1-ium-1-yl]-1-(4-fluorophenyl)ethanone;bromide | 12.01 | (Wang *et al.*, 2013). |
| **C60** | **** | O=C(C1=CC=C(Br)C=C1)C[N+](C2=C3C=CC=C2)=CN3C(C4=CC=CC=C4)C5=CC6=CC=CC=C6O5.[Br-] | 2-[3-[1-benzofuran-2-yl(phenyl)methyl]benzimidazol-1-ium-1-yl]-1-(4-bromophenyl)ethanone;bromide | 3.35 | (Wang *et al.*, 2013). |
| **C61** | **** | O=C(C1=CC=C(OC)C=C1)C[N+](C2=C3C=CC=C2)=CN3C(C4=CC=CC=C4)C5=CC6=CC=CC=C6O5.[Br-] | 2-[3-[1-benzofuran-2-yl(phenyl)methyl]benzimidazol-1-ium-1-yl]-1-(4-methoxyphenyl)ethanone;bromide | 2.29 | (Wang *et al.*, 2013). |
| **C62** | **** | O=C(C1=CC=C(C=CC=C2)C2=C1)C[N+](C3=C4C=CC=C3)=CN4C(C5=CC=CC=C5)C6=CC7=CC=CC=C7O6.[Br-] | 2-[3-[1-benzofuran-2-yl(phenyl)methyl]benzimidazol-1-ium-1-yl]-1-naphthalen-2-ylethanone;bromide | 3.13 | (Wang *et al.*, 2013). |
| **C63** | **** | COC(C=C1)=CC=C1NC(C2=CC=CC=C2C3=NC(C#N)=C(C#N)N3)=O | 2-(4,5-dicyano-1H-imidazol-2-yl)-N-(4-methoxyphenyl)benzamide | 9.2±0.12 | (Malik *et al.*, 2021). |
| **C64** | **** | FC(C=C1)=CC=C1NC(C2=CC=CC=C2C3=NC(C#N)=C(C#N)N3)=O | 2-(4,5-dicyano-1H-imidazol-2-yl)-N-(4-fluorophenyl)benzamide | 8.9±0.11 | (Malik *et al.*, 2021). |
| **C65** | **** | O=C(NC1=CC=CC=C1OC)C2=CC=CC=C2C3=NC(C#N)=C(C#N)N3 | 2-(4,5-dicyano-1H-imidazol-2-yl)-N-(2-methoxyphenyl)benzamide | 9.3±0.37 | (Malik *et al.*, 2021). |
| **C66** | **** | O=C(NC1=CC=CC=C1C)C2=CC=CC=C2C3=NC(C#N)=C(C#N)N3 | 2-(4,5-dicyano-1H-imidazol-2-yl)-N-(o-tolyl)benzamide | 11.6±0.15 | (Malik *et al.*, 2021). |
| **C67** | **** | S=C1N(/N=C/C2=C(O)C=CC=C2)C(C3=CC=C(OC)C=C3)=CN1 | (E)-1-((2-hydroxybenzylidene)amino)-5-(4-methoxyphenyl)-1,3-dihydro-2H-imidazole-2-thione | 1.071±0.1 | (Malik *et al.*, 2021). |
| **C68** | **** | COC1=C(OC)C(OC)=CC(N2C=CN=C2C3=CC=C(OC)C(F)=C3)=C1 | 2-(3′-Fluoro-4′-methoxyphenyl)-1-(3′,4′,5′-trimethoxyphenyl)-1H-imidazole | 0.074 | (Romagnoli *et al.*, 2016). |
| **C69** | **** | COC1=C(OC)C(OC)=CC(N2C=CN=C2C3=CC=C(OCC)C=C3)=C1 | 2-(4′-Ethoxyphenyl)-1-(3′,4′,5′-trimethoxyphenyl)-1H-imidazole | 0.0015 | (Romagnoli *et al.*, 2016). |
| **C70** | **** | COC1=C(OC)C(OC)=CC(N2C=CN=C2C3=CC=C(OCC)C(F)=C3)=C1 | 2-(3′-Fluoro-4′-ethoxyphenyl)-1-(3′,4′,5′-trimethoxyphenyl)-1H-imidazole | 0.0034 | (Romagnoli *et al.*, 2016). |
| **C71** | **** | COC1=C(OC)C(OC)=CC(N2C=CN=C2C3=CC=C(OCC)C(Cl)=C3)=C1 | 2-(3′-Chloro-4′-ethoxyphenyl)-1-(3′,4′,5′-trimethoxyphenyl)-1H-imidazole | 0.0007 | (Romagnoli *et al.*, 2016). |
| **C72** | **** | COC1=C(OC)C(OC)=CC(N2C=CN=C2C3=CC=C(SCC)C=C3)=C1 | 2-(4′-(Ethylthio)phenyl)-1-(3′,4′,5′-trimethoxyphenyl)-1H-imidazole | 0.069 | (Romagnoli *et al.*, 2016). |
| **C73** | **** | CCCOC1=CC=C(C2=NC=CN2C3=CC(OC)=C(OC)C(OC)=C3)C=C1 | 1-(3′,4′,5′-Trimethoxyphenyl)-2-(4′-n-propoxyphenyl)-1H-imidazole | 0.0017 | (Romagnoli *et al.*, 2016). |
| **C74** | **** | COC1=C(OC)C(OC)=CC(N2C=CN=C2C3=CC=C(OC)C=C3)=C1 | 2-(4-methoxyphenyl)-1-(3,4,5-trimethoxyphenyl)-1H-imidazole | 0.23 | (Romagnoli *et al.*, 2016). |
| **C75** | **** | COC1=C(OC)C(OC)=CC(N2C=CN=C2C3=C(C4=CC=CC5=C4C=CC=C5)C=CC=C3)=C1 | 2-(2-(naphthalen-1-yl)phenyl)-1-(3,4,5-trimethoxyphenyl)-1H-imidazole | 0.0046 | (Romagnoli *et al.*, 2016). |
| **C76** | **** | S=C1NC(N2N=NC3=CC=CC=C32)=CN1C4=CC=CC=C4 | 4-(1H-Benzo[d][1,2,3]Triazol-1-yl)-1-Phenyl-1H-Imidazole-2(3H)-Thione | 6.4±0.18 | (Khayyat *et al.*, 2021). |
| **C77** | **** | S=C1NC(N2N=NC3=CC=CC=C32)=CN1C4=CC=C(CC)C=C4 | 4-(1H-Benzo[d][1,2,3]Triazol-1-yl)-1-(4-Ethylphenyl)-1H-Imidazole-2(3H)-Thione | 10.6±0.31 | (Khayyat *et al.*, 2021). |
| **C78** | **** | S=C1NC(N2N=NC3=CC=CC=C32)=CN1C4=CC=C(OC)C=C4 | 4-(1H-Benzo[d][1,2,3]Triazol-1-yl)-1-(4-Methoxyphenyl)-1H-Imidazole-2(3H)-Thione | 2.29±0.07 | (Khayyat *et al.*, 2021). |
| **C79** | **** | S=C1NC(N2N=NC3=CC=CC=C32)=CN1C4=CC=C(O)C=C4 | 4-(1H-Benzo[d][1,2,3]Triazol-1-yl)-1-(4-Hydroxyphenyl)-1H-Imidazole-2(3H)-Thione | 4.45±0.13 | (Khayyat *et al.*, 2021). |
| **C80** | **** | S=C1NC(N2N=NC3=CC=CC=C32)=CN1C4=CC=C(Cl)C=C4 | 4-(1H-Benzo[d][1,2,3]Triazol-1-yl)-1-(4-Chlorophenyl)-1H-Imidazole-2(3H)-Thione | 7.29±0.21 | (Khayyat *et al.*, 2021). |
| **C81** | **** | S=C1NC(N2N=NC3=CC=CC=C32)=CN1C4=CC=CC(Cl)=C4 | 4-(1H-Benzo[d][1,2,3]Triazol-1-yl)-1-(3-Chlorophenyl)-1H-Imidazole-2(3H)-Thione | 2.66±0.08 | (Khayyat *et al.*, 2021). |
| **C82** | **** | S=C1NC(N2N=NC3=CC=CC=C32)=CN1C4=CC=C(Cl)C=C4Cl | 4-(1H-Benzo[d][1,2,3]Triazol-1-yl)-1-(2,4-Dichlorophenyl)-1H-Imidazole-2(3H)-Thione | 3.57±0.16 | (Khayyat *et al.*, 2021). |
| **C83** | **** | O=S(C1=CC=C(N2C=C(N3N=NC4=CC=CC=C43)NC2=S)C=C1)(N)=O | 4-(4-(1H-Benzo[d][1,2,3]Triazol-1-yl)-2-Thioxo-2,3-Dihydro-1H-Imidazol-1-yl)Benzenesulfonamide | 6.1±0.75 | (Khayyat *et al.*, 2021). |
| **C84** | **** | O=C1N(C2=CC(OC)=C(OC)C(OC)=C2)C(C3=CC=C(Br)C=C3)=CN1C(C)=O | 1-Acetyl-1,3-dihydro-3-(3,4,5-trimethoxyphenyl)-4- (4-bromophenyl)-2H-imidazol-2-one | 10.6 | (Xue *et al.*, 2008). |

**Table S2: Protein information for molecular docking protocol**

| **PDB-ID** | **Resolution** | **Organism** | **Molecules** | **Ligand** | **Target** |
| --- | --- | --- | --- | --- | --- |
| **7CHM** | 2.65 Å | Homo Sapiens | Dual specificity protein kinase TTK | 4-(cyclohexylamino)-2-[(2-methoxy-4-morpholin-4-ylcarbonyl-phenyl)amino]-7H-pyrrolo[2,3-d]pyrimidine-5-carbonitrile | Monopolar spindle 1 kinase (MPS1) |
| **3HY3** | 1.80 Å | Homo Sapiens | 5-formyltetrahydrofolate cyclo-ligase | VSNRWTPIADGZQS-GVIHMSOPSA-N  NICKEL (II) ION  MAGNESIUM ION | 5,10-Methenyltetrahydrofolate synthetase (MTHFS) |
| **3PP0** | 2.25 Å | Homo Sapiens | Receptor tyrosine-protein kinase erbB-2 | 2-(Alam and Khan)amino)-5H-pyrrolo[3,2-d]pyrimidin-5-yl]ethoxy}ethanol | HER2 (erbB2). |
| **4ZVM** | 1.97 Å | Homo Sapiens | Ribosyldihydronicotinamide dehydrogenase [quinone] | Flavin-adenine dinucleotide  Doxorubicin  ZINC ION | Quinone reductase 2 (NQO2) |
| **4UDD** | 1.80 Å | Homo Sapiens | Glucocorticoid receptor  Nuclear receptor coactivator 2 | 3-[(3-cholamidopropyl)dimethylammonio]-1-propanesulfonate  Desisobuytyryl ciclesonide  1,2-ethanediol | GR in complex with desisobutyrylciclesonide |
| **5NWH** | 2.60 Å | Homo Sapiens | ADP-sugar pyrophosphatase | 7-[[5-(3,4-dichlorophenyl)-1,3,4-oxadiazol-2-yl]methyl]-1,3-dimethyl-8-piperazin-1-yl-purine-2,6-dione | NUDT5 inhibitor |

**Table S-3:** Molecular interactions attained by the protein ligand docking of compound C6 with 4ZVM & 5NWH

| **Compound** | **PDBID** | **Binding**  **energy** | **No. of H-bonds** | **Amino acid residues** | **Category** | **Type of interactions** | **Distance**  **(Å)** | |
| --- | --- | --- | --- | --- | --- | --- | --- | --- |
| **C6** | **4ZVM** | -10.3 | 0 | GLU193  PHE17  TYR104  PRO192 | Electrostatic  Hydrophobic  Hydrophobic  Hydrophobic | Pi-Anion  Pi-Sigma  Pi-Pi Stacked  Alkyl | 3.88226  2.80224  5.6488  5.41094 | |
|  | **5NWH** | 8.7 | 0 | ARG196  VAL49  VAL49  CYS91  VAL29  PRO86 | Electrostatic  Hydrophobic  Hydrophobic  Other  Hydrophobic  Hydrophobic | Pi-Cation  Pi-Sigma  Pi-Sigma  Pi-Sulfur  Pi-Alkyl  Pi-Alkyl | 4.82409  3.8854  3.69205  5.48025  5.16402  5.33018 |  |

**Table S-4: Molecular interactions attained by the protein ligand docking of compound C10 with PDB ID: 7CHM, 3HY3, 4DDU, 3PPO**

| **Compound** | **PDB ID** | **Binding energy** | **Protein ligand interactions by H-bonding** | | | | **Protein ligand interactions by Van der Waal forces** | | | | |
| --- | --- | --- | --- | --- | --- | --- | --- | --- | --- | --- | --- |
|  |  |  | **No of H-bonds** | **Amino acids** | **Category** | **Type of interactions** | **Distance**  **(Å)** | **Amino acids** | **Category** | **Type of interactions** | **Distance**  **(Å)** |
| **C10** | **7CHM** | -10.5 | 5 | ASP608  SER611  GLN530  ASP674  ILE607 | Hydrogen Bond  Hydrogen Bond  Hydrogen Bond  Hydrogen Bond  Hydrogen Bond | Conventional Hydrogen Bond  Conventional Hydrogen Bond  Conventional Hydrogen Bond  Conventional Hydrogen Bond  Carbon Hydrogen Bond | 2.06949  3.05761  2.75474  2.3491  3.23485 | ILE531  ILE531  ILE607  LEU654  LEU654  MET602  CYS604  MET671  --------  ALA651  PRO673  VAL539 ILE663  VAL539  ALA551  ILE663 | Hydrophobic  Hydrophobic  Hydrophobic  Hydrophobic  Hydrophobic  Other  Other  Other  Hydrophobic  Hydrophobic  Hydrophobic  Hydrophobic  Hydrophobic  Hydrophobic  Hydrophobic  Hydrophobic | Pi-Sigma  Pi-Sigma  Pi-Sigma  Pi-Sigma  Pi-Sigma  Pi-Sulfur  Pi-Sulfur  Pi-Sulfur  Pi-Pi Stacked  Alkyl  Alkyl  Pi-Alkyl  Pi-Alkyl  Pi-Alkyl  Pi-Alkyl  Pi-Alkyl | 3.91859  3.73099  3.92159  3.41013  3.6814  5.2478  5.82669  5.21638  5.00373  4.92157  4.48855  4.95466  4.49143  5.07973  4.08147  4.70116 |
| **C21** | **3HY3** | -9.7 | 1 | TRP109 | Hydrogen Bond | Conventional Hydrogen Bond | 2.28633 | TYR83  TYR83  TRP109  PRO81  MET90  TYR152  TYR153  LYS150  LYS150 | Hydrophobic  Hydrophobic  Hydrophobic  Hydrophobic  Hydrophobic  Hydrophobic  Hydrophobic  Hydrophobic  Hydrophobic | Pi-Pi Stacked  Pi-Pi Stacked  Pi-Pi T-shaped  Alkyl  Alkyl  Pi-Alkyl  Pi-Alkyl  Pi-Alkyl  Pi-Alkyl | 4.16754  4.62507  5.2439  4.61038  4.59813  4.51487  5.3256  4.98386  3.82802 |
| **C21** | **4DDU** | -9.1 | 3 | GLN642  GLN738  PRO637 | Hydrogen Bond  Hydrogen Bond  Hydrogen Bond | Conventional Hydrogen Bond  Conventional Hydrogen Bond  Carbon Hydrogen Bond | 2.54649  2.75662  3.52153 | MET745  TRP557  TYR735 | Other  Hydrophobic  Hydrophobic | Pi-Sulfur  Pi-Pi Stacked  Pi-Pi Stacked | 5.24629  5.5483  4.22602 |
| **C76** | **3PPO** | -10.3 | 1 | LYS753 | Hydrogen Bond | Conventional Hydrogen Bond | 2.58186 | LEU726  VAL734  VAL734  THR798  LEU852  ALA751  LEU852  ALA751  VAL734  ALA751  LYS753  LYS753  LEU796 | Hydrophobic  Hydrophobic  Hydrophobic  Hydrophobic  Hydrophobic  Hydrophobic  Hydrophobic  Hydrophobic  Hydrophobic  Hydrophobic  Hydrophobic  Hydrophobic  Hydrophobic | Pi-Sigma  Pi-Sigma  Pi-Sigma  Pi-Sigma  Pi-Sigma  Pi-Alkyl  Pi-Alkyl  Pi-Alkyl  Pi-Alkyl  Pi-Alkyl  Pi-Alkyl  Pi-Alkyl  Pi-Alkyl | 3.72007  3.46447  3.95536  3.43992  3.54125  4.38572  5.18474  4.89583  5.11592  4.60183  4.26135  4.8689  5.11497 |

**Table S5: The RMSD calculated value between the original co-crystal position and docked poses**

| **Protein (PDB ID)** | **RMSD value** |
| --- | --- |
| 4ZVM | 0.00 |
| 5NWH | 0.00 |
| 7CHM | 0.456 |
| 3HY3 | 0.00 |
| 4UUD | 0.00 |
| 3PP0 | 0.940 |

**Table S6: Quantum chemical parameters based upon DFT computations**

| Parameters | C10 |
| --- | --- |
| E_HOMO_(eV) | -0.21971 |
| E_lumo_(eV) | -0.08866 |
| Energy gap ΔE (ev) | 0.13104 |
| Ionization potential (I=-E_HOMO_) | 0.21971 |
| Electron affinity (A=-E_LUMO_) | 0.08866 |
| Electronegativity (χ=$\frac{\mathbf{(I+A)}}{\mathbf{2}}$) (ev) | 0.30836 |
| Chemical potential (µ= -$\frac{\mathbf{(I+A)}}{\mathbf{2}}$) (eV) | -0.30836 |
| Chemical hardness (η=$\frac{\mathbf{(I-A)}}{\mathbf{2}}$) (eV) | 0.0655 |
| Chemical softness (S= $\frac{\boldsymbol{1}}{\mathbf{2}\boldsymbol{\eta}}$) (eV) | 7.6336 |
| Electrophilicity index (ω= $\frac{\boldsymbol{\mu2}}{\mathbf{2}\boldsymbol{\eta}}$) (eV) | 0.725 |
| Nucleophilicity index (N= $\frac{\mathbf{1}}{\boldsymbol{\omega}}$) (eV) | 1.379 |
| Maximum Charger transfer  index (ΔN_max_=$\frac{\boldsymbol{-\mu}}{\boldsymbol{\eta}}$) (eV) | 4.7078 |
| Dipole moment (Debye) | 7.126807 |
